# Human-Derived Disturbance Estimation and Compensation (DEC) Method Lends Itself to a Modular Sensorimotor Control in a Humanoid Robot

**DOI:** 10.3389/fnbot.2017.00049

**Published:** 2017-09-08

**Authors:** Vittorio Lippi, Thomas Mergner

**Affiliations:** Neurology, University Clinics of Freiburg Freiburg, Germany

**Keywords:** sensory-motor system, humans, neuromechanical modeling, modular control architecture, humanoid robot experiments

## Abstract

The high complexity of the human posture and movement control system represents challenges for diagnosis, therapy, and rehabilitation of neurological patients. We envisage that engineering-inspired, model-based approaches will help to deal with the high complexity of the human posture control system. Since the methods of system identification and parameter estimation are limited to systems with only a few DoF, our laboratory proposes a heuristic approach that step-by-step increases complexity when creating a hypothetical human-derived control systems in humanoid robots. This system is then compared with the human control in the same test bed, a posture control laboratory. The human-derived control builds upon the identified disturbance estimation and compensation (DEC) mechanism, whose main principle is to support execution of commanded poses or movements by compensating for external or self-produced disturbances such as gravity effects. In previous robotic implementation, up to 3 interconnected DEC control modules were used in modular control architectures separately for the sagittal plane or the frontal body plane and successfully passed balancing and movement tests. In this study we hypothesized that conflict-free movement coordination between the robot's sagittal and frontal body planes emerges simply from the physical embodiment, not necessarily requiring a full body control. Experiments were performed in the 14 DoF robot Lucy Posturob (i) demonstrating that the mechanical coupling from the robot's body suffices to coordinate the controls in the two planes when the robot produces movements and balancing responses in the intermediate plane, (ii) providing quantitative characterization of the interaction dynamics between body planes including frequency response functions (FRFs), as they are used in human postural control analysis, and (iii) witnessing postural and control stability when all DoFs are challenged together with the emergence of inter-segmental coordination in squatting movements. These findings represent an important step toward controlling in the robot in future more complex sensorimotor functions such as walking.

## Introduction

### Human posture control modeling and neurorobotics

Human postural control attracts considerable interest in healthcare research worldwide for reasons such as “fall of the elderly” and neurological impairments such as ataxia in cerebellar patients or deficient movement control in Parkinson's disease. Developing *model-based* diagnostics as well as therapeutic and rehabilitative interventions is an important aim of this research. Engineering-inspired approaches to model the human sensorimotor control and its failures have a long tradition (e.g., Nashner, 1972; Hajos and Kirchner, 1984; Johansson et al., 1988; Kuo, 1995; Fitzpatrick et al., 1996). These approaches often address *reactive* postural responses to well-controlled external stimuli, which lend themselves to system identification, whereas the exact input for voluntary movements is generally unknown. Two of the following recent modeling approaches were especially influential, yet their clinical application can often prove to be problematic.

One modeling approach used system state estimation based on multi-sensory integration under noise optimization principles. This approach builds on biological textbook knowledge on human sensors and anthropometrics and, based on this knowledge and engineering principles, analyzes the human posture control using noise optimizing principles (van der Kooij et al., 2001; Kuo, 2005). For example, it predicts that, whenever possible, posture control preferentially uses proprioceptive rather than vestibular information, as the vestibular information is the one containing more noise. These models allow general predictions on human preferences for certain sensory environments and may define which sensory deficit in patients tends to increase the danger of falling. However, poor correspondence of these models with human neurophysiology and anatomy restricts its clinical usefulness for diagnosing more specific posture control problems of an individual patient.

The other modeling approach used time series or frequency domain data gathered from posture control experiments in humans to establish the simplest model compatible with known human physiology and anatomy that would allow reproduction of the data with identified model parameters. Best known is the independent channel (IC) model of Peterka (2002) for single inverted pendulum (SIP) scenarios. It identifies sensory weights depending on the proper selection and interpretation of results using different stimuli and test conditions. From extensions of this model to more complex scenarios it was concluded that available engineering methods are in principle capable of arriving at multi-segmental control models. However, the increasing model complexity and the proliferation of parameters tend to reduce the chance of unequivocally identifying the control parameters in health and disease (Mergner and Peterka, 2017). The problem is aggravated by non-linearities of the human control system (described as detection thresholds in Maurer et al., 2006).

As such, there currently exists a dilemma as to which methodology can be used to establish model-based diagnostics and therapeutic or rehabilitative interventions for patients with impaired postural control. The goal of the present study is to contribute to a *heuristic* solution. By this we mean a practicable solution that proceeds from an established model of the human control in the SIP scenario and uses plausible arguments and steps for its extension to more degrees of freedom (DoFs). To evaluate the appropriateness of these steps, we test their effects in special-purpose humanoid robots with human-inspired anthropometrics, sensors, and actuators. In this way a “real world” challenge is imposed which accounts for noisy and inaccurate sensors and non-ideal actuation and mechanics. For as much correspondence as possible to the human situation, these tests are performed in the same testbed that is also used for the human subjects, i.e., in a human posture control laboratory. As described below, first steps in this heuristic approach have successfully been performed. Point of departure was the “disturbance estimation and compensation” (DEC) model for the SIP scenario, which shares basic similarities with the IC model (Mergner et al., 2003; Maurer et al., 2006; Mergner, 2010). While several extension steps have been described previously (see section Previous and Current Steps in the Heuristic Approach), here we report on an extension to a 14 DoF robot and examine whether a modular architecture consisting of a net of DEC controls in the robot's sagittal plane cooperates in a conflict-free way with a corresponding but independent net of DEC controls in the frontal plane during both disturbance compensation and commanded (“voluntary”) movements.

The following subsections aim to combine state of the art human sensorimotor issues with recent robotics issues dealing with posture control. Here and in later sections, we include brief descriptions on the current state of our bottom-up implementation of a human-like postural control in robots. First, we briefly review the biological basis of the DEC model (section Main Features of the Human DEC Model), then consider related issues in neuroscience and robotics (section Modular Control Issues in Neuroscience and Robotics), and finally present a list of the previously performed steps in our neurorobotics approach and the new steps taken in this study (section Previous and Current Steps in the Heuristic Approach).

### Main features of the human DEC model

**Figure 1:**
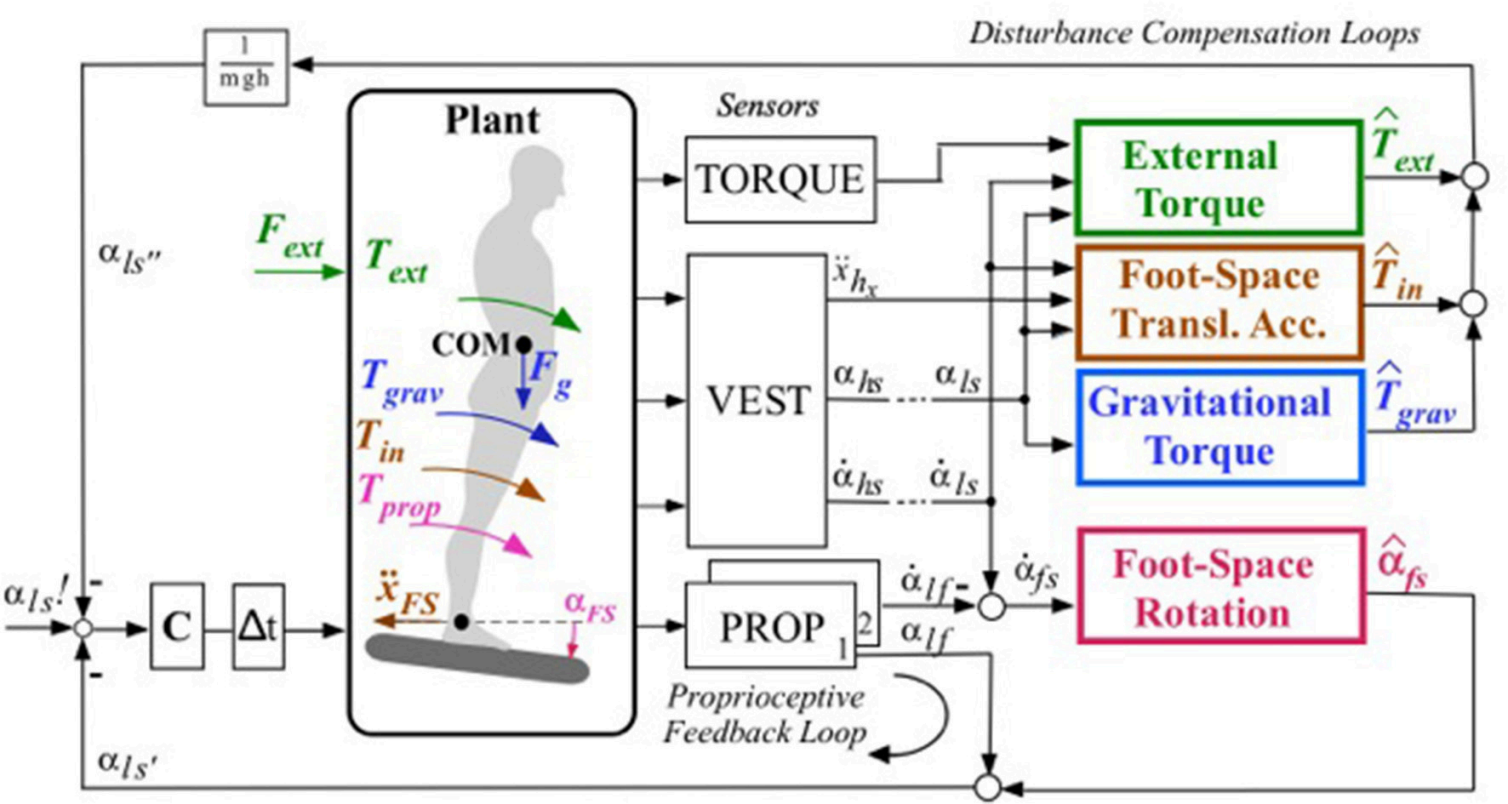
Schema of DEC control of the ankle joint in the single inverted pendulum, SIP, scenario with support surface tilt forward (this model is the basis for the modular control architexture in multi-DoF systems). Four external disturbances (external force *F*_*ext*_, gravitational force *F*_*g*_, foot-space rotation α_*FS*_ and foot-space linear acceleration ẍ_*FS*_) give rise to the joint torques (*T*_*ext*_, *T*_*grav*_, *T*_*prop*_, and *T*_*in*(=*inertial*)_, respectively). Sensors (VEST, vestibular; PROP, proprioceptive; TORQUE) are actually networks that combine signals from a variety of transducers (e.g., 3 VEST sensors result from vestibular canal-otolith intractions; Mergner et al., 2009) to yield information on the following physical quantities: ẍ_*hx*_, head acceleration in x (sagittal forward) direction, and α_*hs*_ and $\dot{\alpha }$_*hs*_, head-in-space angle and angular velocity (dashes indicate proprioceptive coordinate transformation to lower body segments, see Figure 2; here they point out that in the SIP scenario these vestibular signals refer to the whole body above the anke joints, includinging the legs, i.e., α_*ls*_ and $\dot{\alpha }$_*ls*_); α_*lf*_ and $\dot{\alpha }$_*lf*_ represent leg-to-foot angle and angular velocity, respectively. Colored boxes derive disturbance estimates (indicated by hats) from the reported physical quantities. Variable foot-in-space angular velocity ($\dot{\alpha }$_*fs*_) is time-integrated leading to the estimate $\hat{\alpha }$_*fs*_ of foot-in-space position; by means of this signal the proprioceptive signal α_*lf*_ is being “upgraded” (transformed) into space coordinates ($\alpha_{ls^{\prime }}$). C, neural controler; Δt, lumped neural time delay; α_*ls*_*!*, desired leg-space angle. Box 1/*mgh* transforms torque into an equivalent of an angle. Passive stiffness and its modulation (in humans achieved by muscle co-contration) is omited here for simplicity (compare Ott et al., 2016 as to its role for control stability). In current versions of the DEC concept, the input may be varied in three ways depending on the task. The task shown in this figure is to reach and maintain a given orientation of the supported body segment, here the leg segment (in fixed alignment with upper body), in space (α_*ls*_*!*). In situations where the body COM changes with the body configuration or load distribution, the task refers to the body COM in space (*bs*; α_*bs*_*!*) and requires that the current COM location is taken into account in the control. This has been experimentally tested and modeled for human responses in ankle and hip joints to support surface tilts in the sagittal plane (Hettich et al., 2014). As an intuitive third possibility, one may want to reach and maintain a given joint angle, which in the scenario of this figure would mean to command the leg-foot angle α_*lf*_*!* as input and to neglect estimate $\hat{\alpha }$_*fs*_.

Both the DEC model and the clearly simpler IC model can describe results from the same protocols for human balance control experiments. The IC model is a linear model that analytically describes human sway responses to support surface tilt in the frequency domain based on a control by proprioceptive, vestibular, and visual feedback channels (Peterka, 2002). The model allows for identification of important features of the human postural control system, the most important ones being time delays in the order of 100–200 ms associated with low loop gain and, as a consequence, soft mechanical compliance, and low energy consumption. It also identifies *sensory reweighting*, meaning that humans adjust their use of sensory information to changes in perturbation amplitude and modality. While the IC model describes this feature by using different sets of control parameters, the DEC model (Figure 1) is able to predictively describe the data from various experimental conditions with one set of control parameters. The DEC model contains *synthetic* and *holistic* features-synthetic in the sense that it uses disturbance estimations inspired from studies on multisensory fusions in human self-motion perception (Mergner et al., 1997; Mergner and Rosemeier, 1998), holistic in the sense that it also integrates the control of *movements* in a single structure.

The DEC model extends upon the engineering concept of the servo control by negative feedback (see Wiener, 1948, for the early developments of the concept). In neurology, Merton (1953) used this concept to explain the role of the muscle stretch reflex for the control of posture and movements. He posits that a PD-controller adjusts the force of the muscles so as to produce the desired pose or movement. This would be achieved through negative feedback from proprioceptive sensors, i.e., by feeding the controller with the difference between the desired and the sensed joint angle. However, later researchers considered this concept to be problematic. One reason was that the biological time delay in the feedback loop does not allow for stable performance when large external disturbances such as gravity require high loop gains (see McIntyre and Bizzi, 1993). The DEC model overcomes this problem by estimating the external disturbances and commanding the servo to produce the extra force required for their compensation. The underlying principle is known in control theory under different names, e.g., “feed forward disturbance correction” (Roffel and Betlem, 2006); similar (Luecke and McGuire, 1968; Zhong et al., 2012); in German consistently “Störgrößenaufschaltung;” Bleisteiner et al., 1961). The DEC mechanisms operate *context*- and *intent*-dependently (Mergner, 2010). In the terminology of sensorimotor physiology, we subsume them in contradistinction to “short latency reflexes” under “long latency reflexes,” which are known to be modifiable by higher brain centers such as the cerebral cortex (see Pruszynski and Scott, 2012). Disturbance compensation in the DEC is thought to arise reactively with unforeseen external disturbances as well as in the form of predicted-sensory estimates, issued by higher brain centers with foreseen external or self-produced disturbances. Such a “proactive” compensation is assumed to be advantageous compared to a “reactive” one due to short central time delays and absence of sensory noise, leading to more stable control (see Maurer et al., 2006). Noticeably, several reactive and proactive compensatory actions may arise simultaneously, even during voluntary movements (i.e., the “superposition law” applies despite the non-linarities in the disturbance estimates).

As previously explained in more detail (Maurer et al., 2006), humans use several sensory inputs (proprioceptive, vestibular, visual, haptic contact, torque, and pressure) for posture control. They combine this information in various ways to estimate physical variables such as joint angle and angular velocity from muscle spindle, skin receptor, tendon organ, and torque inputs, as well as head linear and angular acceleration from vestibular otolith and semicircular canal inputs, for example. From the estimated physical variables, the DEC model then derives estimates of four classes of disturbances that may influence the torque acting on the skeletal joints (colored boxes in Figure 1): (1) *Rotation* and (2) *translation* of the support (be this a supporting body segment or an external support), (3) *gravity and other field forces*, and (4) *contact forces* (“external torque”). As depicted in Figure 1 (which refers to the SIP scenario), in the case of balancing, the input of the model is the desired angle of the leg segment (here for SIP) in space α_*ls*_*!*, a signal that is thought to stem from higher brain centers including the cerebral cortex. The cortex builds a coherent and continuously updated sensorimotor model of the body as a whole from many input sources. This is often referred to as “body schema:” a conception from neurology that has found its way into humanoid robotics (Morasso, 2013). In the DEC model, noticeably, both the cortical movement commands from the body schema and the conscious perception of the produced movement almost exclusively reflect kinematics. Signals related to stereotypically occurring forces from gravity, link inertia, inter-link coupling forces, etc. are hidden, so to speak, because they are compensated by the DEC mechanism (details in Mergner, 2010).

In this form, the DEC concept qualified for a modular control architecture in scenarios with >1 DoF (see Section Previous and Current Steps in the Heuristic Approach). Modular control as a concept has attracted considerable interest earlier in neuroscience and robotics. The following section points out differences to the present concept.

### Modular control issues in neuroscience and robotics

In the DEC concept, a modular control by two DEC modules has been used for the modeling of human ankle joint and hip joint responses to support surface tilt and for mimicking the responses in a robot (Hettich et al., 2014). Further work supported this idea of a modular control, for example when we added a third DEC control in a robot for proactive squatting in the knee joints during reactive balancing (Ott et al., 2016 and https://www.youtube.com/watch?v=3ALCTMW3Ei4).

Generally, modularity is often applied at the level of kinematics, as is the case in neuroscience with “movement synergies,” where each control module commands a subset of the controlled DoFs. Specifically, a kinematic synergy is often defined as a function mapping a scalar value to several DoFs. Such synergies are often used to reduce the dimensionality of the control problem with the result that the number of controlled synergies is smaller than the total number of DoFs. The principle has not only inspired research of human walking (e.g., Ivanenko et al., 2003), but also various fields of humanoid control (Hauser et al., 2007). Noticeably, however, the number of required synergies in complex movements may reach the number of DoFs. This may happen for example when movement control is organized in terms of eigenmovements (Alexandrov et al., 2017) where the aim is to free the control from coupling forces stemming from other movements. Certain synergies studied in humans, such as torque synergies, are so far largely neglected in robotics. Another related concept is that of motor primitives, originally meaning basic kinematic, dynamic, or muscular building blocks of movements arising at neuronal levels (Flash and Hochner, 2005). Generally, motor primitives can be combined in several ways, i.e., to act simultaneously by summing them or to obtain superposition of effects, as is the case with synergies, or serial effects when using them in a sequence, as with predefined trajectories or velocity profiles. The concept of motor primitives has found extensive application in robotics for learning of motor tasks (Schaal et al., 2003). Some implementations of modularity are not only advantageous in that they reduce complexity in control design, but also in increasing control robustness (in case one module fails, remaining modules can take over).

**Figure 2:**
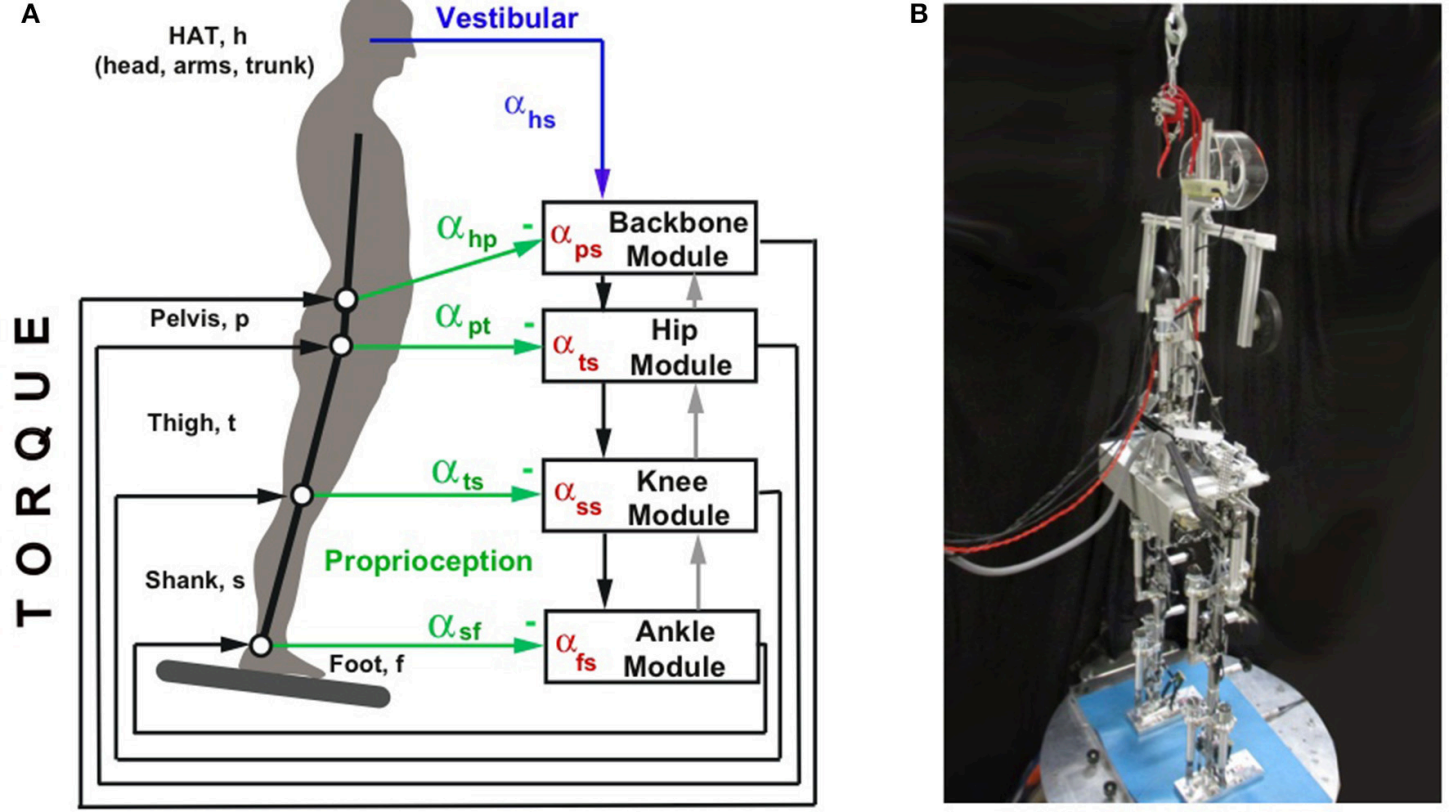
**(A)** Schematic representation of the interconnections between the DEC control modules, in terms of down-channeling and up-channeling of sensory information in a given body plane (here sagittal plane; compare Figure 4 for analogies in the frontal plane). Shown by solid downward arrows is the distribution of the vestibular signal “head angle with respect to the gravitational vertical” (α_*hs*_). Analog distributions hold for the angular velocity signal $\dot{\alpha }$_*hs*_ and the head linear acceleration signal in x (sagittal forward) direction, ẍ_*Hx*_. The gray upward arrows show the up-channeling of processed signals, representing the causal physical interactions in a stack of superimposed body segments. For example, up channeling of $\hat{\alpha }$_*fs*_ from the ankle module to the knee module and fusing it with the proprioceptive α_*lf*_ signal may be used in the knee module as a predictor of α_*hs*_ (a mechanism that bypases the sensory feedback chain and increases control stability). This up-channeling is especially effective if the support surface is stationary, whereby $\hat{\alpha }$_*fs*_ becomes subthreshold (through a velocity threshold) and with it the effect of the particularly noisy vestibular $\dot{\alpha }$_*hs*_ signal; see Mergner et al., 2009). **(B)** Picture of Lucy Posturob (from right and behind) balancing on the motion platform in the human posture control laboratory.

Compared to these modularity concepts, the architecture of combining DEC modules is clearly distinct. Here, each DoF of the human skeletal system is controlled by one DEC module. This even applies to situations where two or more modules do the same job and conceptually may be viewed as one module (e.g., during sagittal body sway about the two ankle joints with aligned axes and the body weight equally distributed on both feet). Each module determines the torque to be applied to the controlled DoF. The desired trajectory is specified as an input to each module. All modules have essentially the same structure and have no internal model of the whole system. Yet, the modules operate not completely independently of each other, because they exchange sensory information through coordinate transformation across the joints that interconnect the body segments (Figure 2A). Compared to the above examples of modular control, the DEC model can be defined as a low level control system that takes care of the fundamental task of posture control for undisturbed motor execution and acts at the level of joint kinematics. Coordination between different joints may emerge from the interaction between the modules and the body mechanics under task, even if no kinematic synergy is explicitly specified. For example, hip-ankle coordination in the experiments of Hettich et al. (2014) emerged from the tasks for the ankle and hip controllers to maintain the body COM over the feet as base of support and the trunk upright, respectively.

Further aspects will be considered in section Discussion.

### Previous and current steps in the heuristic approach

In previous works, the DEC model was subjected to “real world” tests, separately for the sagittal and the frontal plane. The tests were performed with humanoid robots equipped with human-inspired sensors, actuation, and anthropomorphic properties in the human posture control laboratory. Robot responses to support surface tilt in the sagittal plane were successfully compared to human responses once in a SIP scenario (Mergner et al., 2009) and later in a DIP scenario (Hettich et al., 2014). The latter study also showed that it sufficed to use interconnected DEC control modules for the ankle and the hip joints in order to simulate the human responses in the robot (for simplification, the joints on both sides were mechanically coupled). Interestingly, the ankle-hip coordination emerged from the interconnection of the two modules (see section Modular Control Issues in Neuroscience and Robotics). Subsequent experiments focused on a transfer of DEC to the frontal plane (14 DoF robot; Lippi et al., 2016) and the use of the knee joints to test the control of squatting movements in the sagittal plane. To this end, realization of the control in Simulink (The MathWorks Inc., Natick, USA) and using force controlled actuation allowed us to transfer the DEC control to the DLR robot TORO for comparison with a fully model-based control (Ott et al., 2016 and https://www.youtube.com/watch?v=3ALCTMW3Ei4).

This study investigates whether the networks of DEC modules previously controlling the robots' sagittal plane and frontal plane separately would adequately cooperate when combined during movements in intermediate planes. Specifically, we asked whether the physical linkages given by the robot's body suffice to guarantee adequate cooperation between the sensorimotor controls, or whether a supervising full body model or some other form of software linkage between the two networks would be required. The experiments are performed in a robot with 14 DoFs (Lucy Posturob, Figure 2B) using one and the same set of control parameters for non-trivial scenarios that draw on the scalability of the DEC modular control. The tests included both voluntary movements and balancing responses to passive body motions in an intermediate plane. Postural and control stability across all DoF of the robot were tested when the robot performed squatting movements in the knee joints, evoking the emergence of inter-segmental coordination in the ankle and hip joints. Further experiments aimed to provide a quantitative characterization of the robot's balancing responses in terms of FRFs (as they are used in human postural control analyses) and tested postural stability when increasing frequency of voluntary movements simultaneously in the sagittal and frontal body planes up to the performance limits.

## Methods—robotic platform and experimental procedures

### Humanoid robot Lucy Posturob

**Figure 3:**
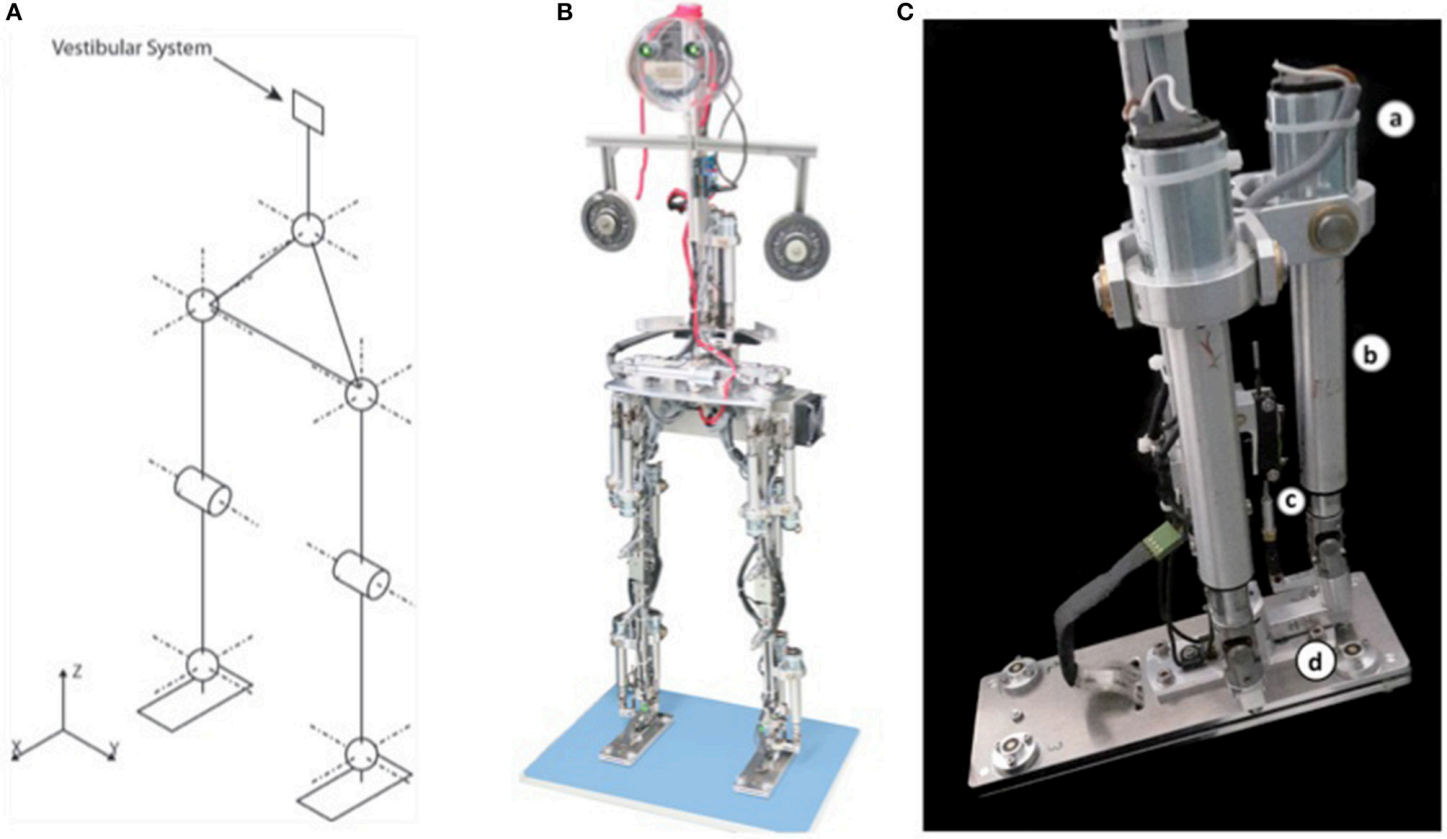
Details of the humanoid robot Lucy. **(A)** Scheme of the 14 degrees of freedom. **(B)** Picture of Lucy Posturob (arms represented by weights; vestibular system is located in head; no visual system is currently provided by the eyes; electronics for actuation is contained in the pelvis). **(C)** Details of foot with actuation (a, DC motor; b, spindle; c, enconder; d, load cell as torque sensor). Actuation is the same in all DoFs.

The Posturob III robot, *Lucy Posturob* (details in Figure 3), was constructed as a humanoid of 1.5 m body height and ~20 kg body weight with anthropometric parameters inspired by Winter (1990). Its body consists of the upper body (HAT, for head, arms, and trunk), pelvis, the two thighs, shanks (lower leg), and foot segments, all made of aluminum and interconnected by hinge joints. The total of 14 DoF (Figures 3A,B) comprise 2 DoF per ankle joint, 1 per knee, 3 per hip, and 2 DoF for the lower vertebral column (“lumped” DoFs across the vertebrae of the back bone).

Using an analog acquisition board, a PC read from mechatronic sensors the signals joint torque, joint angular position and velocity, and the anterior-posterior and medio-lateral pressure distribution under each foot (not used in the present experiments). The same computer also read via USB the signals from a custom-made human-inspired artificial vestibular system (Mergner et al., 2009). The DEC control model was implemented and executed as a compiled Simulink model (Real-Time Windows Target, The MathWorks Inc., Natick, USA). The control system worked at 200 Hz. The delay of the system as a whole is estimated to be 20 ms (which corresponds approximately to the value to which we adjusted all sensory signals for correct time-matched interactions in the disturbance estimations). This delay is, admittedly, shorter that the lumped delays identified in humans (see section Main Features of the Human DEC Model). However, additional features have to be added to the DEC control modules in the Lucy robot before we can aim to repeat some of our experiments with more human-inspired time delays (as in Hettich et al., 2014).

The vestibular sensor was fixed to the robot's HAT segment. A bio-inspired algorithm (Mergner et al., 2009) fuses the inputs from 3 accelerometers and 3 gyrometers to estimate (i) HAT angular velocity in space, (ii) HAT angle with respect to the gravitational vertical, and (iii) linear acceleration of the upper HAT end representing head position (compare Figure 3A). Joint actuation was achieved by DC electric motors (part *a* in Figure 3C) that rigidly interconnected the two links by a screw/spindle system (part *b*). The spindle drive transformed the rotational movement of the motors into a lever movement, as measured by a linear potentiometer, part c, for producing joint angle. An inner torque control loop was implemented on the on-board robot electronics, receiving the torque command signal from the higher-level bio-inspired DEC algorithms on the PC. The force sensor, part d in Figure 3C, measured the tangential force acting on the joint in order to compute the joint torque signal used (i) in the DEC control loop and (ii) in the on-board control of the torque.

### Disturbance estimation in multiple DoF system

The four estimated disturbances in a DEC control module are not directly available as sensory inputs and are hence reconstructed through inter-sensory interactions. The sensory inputs used in these and our previous robot experiments with the DEC model are from the vestibular system, joint proprioception and joint torque (compare Figure 1). Currently unconsidered are visual self-motion cues for balancing (see Assländer et al., 2015), and foot pressure cues.

In the multi-segment human body, the sensor fusions for the disturbance estimates use signals from both local sensors (proprioceptive joint angle and angular velocity sensors and torque sensors) and remote (e.g., vestibular) sensors. The vestibular signals in Lucy are conveyed to the control modules through down-channeling from their source in the head, as schematically shown in Figure 2. Additional information is required for the disturbance estimations concerning mass and inertia distribution of the supported body segments with respect to the supporting joint, as described for the SIP scenario in Mergner (2010). To further account for momentary changes of these parameters in the multi-segment system, Lippi et al. (2013) provided a general description of the down-channeling of the processed sensory information. In particular, for the two disturbance estimators of support rotation and gravitational torque, $\hat{\alpha }$_*fs*_ and $\hat{T}$_*grav*_ (see Figure 1), the COM location of all segments above a given supporting joint is calculated step-wise downwards. This allows for treating the COMs of all supported segments in a given joint as if they were the COM of a single-segment body. For example, with sagittal support surface tilts the hip joints compensate the gravity impact from any upper body lean, while the ankle joints are compensating the gravity effect due to the lean of the whole body COM. This example also shows that, while the action of a control module is local, the estimated disturbances represent global effects acting on the body. For the *support surface translation estimates* $\hat{T}$_*in*_, the simplification by down-channeling concerns the joint torque produced by the combined inertial forces exerted by all upper body segments, while for the *contact force estimates* $\hat{T}$_*ext*_ it is the torque produced by combined forces having impact on the supported segments. The sensor fusions used to reconstruct these global variables are distributed among modules and are based on signal exchanges between modules.

The present study explains the generalized concepts of disturbance estimations with down and up channeling between modules (Figure 2) in more detail. It is worth noting that an advantage of using up-channeling is that the information of the physical variables conveyed upward are already processed at a lower level. This is especially relevant for the estimate of support surface tilt in the ankle joint. Running the input velocity signal through a threshold reduces noise of this estimate (see below). The up-channeling of this signal is used in upper segments, instead of the local input signals in these segments, which carry more noise. This up-channeled foot(support)-in-space signal contributes to the computation of the variables controlled by the servo loops of the higher modules. Module inputs and outputs are shown in Table 1. Included are also the inputs coming directly from the sensory system (e.g., joint angle). These sensor fusions are described here in more detail than before, using a generalized notation that allows for an arbitrary number of DoFs.

**Table 1:** Signals and parameters used in the sensor fusion process.

**Signal**	**Symbol**	**Description**
Body in space sway, up-channeled	$\hat{\alpha_{bs}^{n}}$	Angle sway of the COM of all the segments above the controlled link, obtained using the upchanneled $\hat{\alpha_{fs}^{n}}$ signal. It can be used in the servo loop as controlled variable
Body in space sway, down-channeled	$\overset{⌣}{\alpha_{bs}^{n}}$	Angle sway of the COM of all the segments above the controlled link, obtained using the down-channeled $\overset{⌣}{\alpha_{fs}^{n}}$signal. It is used to compute external disturbances.
Desired value for the controlled variable	$\alpha_{lf}^{n}!$, $\alpha_{bs}^{n}!$, $\alpha_{ls}^{n}!$	Reference for joint angle (lf), body COM orientation in space (bs) and link orientation in space (ls)
Proprioceptive input	$\alpha_{lf}^{n}$	The angular position of the controlled joint. Used to compute $\overset{⌣}{\alpha_{fs}^{n}}$ and $\hat{\alpha_{ls}^{n}}$. It can be used in the servo loop as controlled variable.
Foothold link orientation in space, downchanneled	$\overset{⌣}{\alpha_{fs}^{n}}$	Orientation in space of the link supporting the controlled joint, it is used to compute $\overset{⌣}{\alpha_{bs}^{n}}$and it is passed to the underlying module as $\overset{⌣}{\alpha_{ls}^{n}}$.
Foothold link orientation in space, up-channeled	$\hat{\alpha_{fs}^{n}}$	In the module controlling the support joint it is computed using ^⌣^$\overset{⌣}{{\dot{\alpha }}_{fs}^{n}}$ as shown in Equation (1). In the other modules it is up-channeled from the underlying module as $\hat{\alpha_{ls}^{n}}$.
Controlled link in space rotation speed, down-channeled.	$\overset{⌣}{{\dot{\alpha }}_{fs}^{n}}$	It is down-channeled and used for the computation of $\hat{\alpha_{fs}^{0}}$.
		
Controlled link in space orientation down-channeled.	$\hat{\alpha_{ls}^{n}}$	Angle sway of the COM of all the segments above the controlled link. It is used to compute external disturbances.
Controlled link in space orientation up-channeled.	$\hat{\alpha_{ls}^{n}}$	It can be used in the servo loop as controlled variable
Center of mass position in the controlled plane	$\overset{⌣}{COM^{n}}$	Position in space of the center of mass of all the links above the *n* th joint. It is down-channeled so that in each module it can be updated to take in account the controlled body segment.
Mass of all the segments above the controlled joint	$m_{up}^{n}$	This parameter is down-channeled. Each module is updating it adding the mass of the controlled link, that is an internal parameter
Moment of inertia of all the segments above the controlled joint	$J_{up}^{n}$	The moment of inertia is down-channeled and updated in each module on the basis of the internal parameters describing the controlled link and the configuration of the body.

#### Support surface tilt

In the SIP scenario of Figure 1, an estimate of support surface tilt is obtained by combining a vestibular derived leg-in-space signal and a local ankle proprioceptive leg-on-foot signal. Specifically, the signal used to compensate the support surface rotation is derived from the rotation speed of the foot in space, when the foot is in firm contact with the support surface $\dot{\alpha_{fs}^{1}}$.

(1) $$\hat{\alpha_{fs}^{1}}=\int_{0}^{t}\rho ({\hat{\alpha }}_{fs}^{{}^{{}^{\cdot }}1})-k{\hat{\alpha }}_{fs}^{1}\;d\tau$$

In the generalized notation used, the pedix *fs* stands for *foothold in space*, i.e., the orientation in space of the link under the controlled joint, which represents an extension of the variable *foot in space*. Similarly, in the following the variables *ls* for *link in space* and *lf* for *link to foothold* denote the generalization of the variables *leg in space* and *leg to foot* as used in the SIP and the DIP (double inverted pendulum) case before (Mergner, 2010; Hettich et al., 2013; Lippi et al., 2013). This notation now allows for a description of the sensor fusion process with a generic number of modules, where the index *n* represents the position of the controlled joint (*n* = 1 is the ankle, *n* = 2 the knee, etc.). For specific cases, a notation referring to the names of body segments can be used for simplicity, as shown in Figure 2A where *ts* for example means *thigh in space*. All the signals in (1) are estimates, with the hat denoting here the up-channeled estimate, ρ(·) is the threshold function and *t* the current time. The integration is leaky (modulated by the term *k*).

Given the threshold Θ >0, the function ρ(·) is defined as

(2) $$\rho \left(\alpha \right)=\{\;\begin{array}{c} \alpha +\theta \;\alpha \leq -\theta \\ 0,\;-\theta <\alpha <\theta \;\\ \alpha -\theta \;\alpha \geq \theta \end{array}$$

The value of Θ is a parameter of the control module. The presence of the threshold function ρ(·) introduces a gain non-linearity in the balance behavior (i.e., larger support surface tilts are more compensated than smaller ones), as previously observed in human experiments (e.g., Hettich et al., 2014).

The estimate of support surface tilt from Equation (1) is used to reconstruct the orientation in space of the link above the controlled joint $\hat{\alpha_{ls}^{n}}$ inside each module. The signal is up-channeled and fused with the proprioceptive input throughout all the modules of a given body plane. The resulting value in the nth module is

(3) $$\hat{\alpha_{ls}^{n}}=\hat{\alpha_{fs}^{1}}+\sum_{k=1}^{n}\alpha_{lf}^{k}$$

This is different with the other three sensory disturbance estimates (below), in that these are based on the down-channeled position. This scheme reproduces human-like responses in robot experiments and model simulations. However, the interactions between up-channeled and down-channeled signals in the general case are still under research.

#### Field forces such as gravity

The gravity torque *T*_*g*_ in the general case is calculated by

(4) $$T_{g}=m_{up}^{n}gCOM_{x}^{n}$$

where $COM_{x}^{n}$ is the horizontal component of the position of the center of mass ${\mathrm{COM}}_{\;}^{\text{n}}$ of all the segments above the controlled joint. The estimated ${\mathrm{COM}}_{\;}^{\text{n}}$ is computed performing the weighted sum

(5) $${\overset{⌣}{\mathrm{COM}}}^{n}=\frac{\left({\overset{⌣}{\mathrm{COM}}}^{n+1}+L^{n}\left[\begin{array}{c} \cos(\overset{⌣}{\alpha_{ls}^{n}})\\ \sin(\overset{⌣}{\alpha_{ls}^{n}}) \end{array}\right]\right)m_{up}^{n+1}+h^{n}\cos(\overset{⌣}{\alpha_{ls}^{n}})m_{\;}^{n}}{m_{up}^{n}}$$

where *L*^*n*^ is the length of the link controlled by the joint and *h*^*n*^ is the distance of the COM of the nth link from the nth joint, $m_{\;}^{n}$ is the mass of the nth link, and $m_{up}^{n}$ the total mass of all the links above the nth joint. The inverted hat denotes estimators based on down-channeled signals. The modules can be set to use $\overset{⌣}{{COM}_{x}^{n}}$ or to apply a small angle approximation as done in previous experiments (Mergner, 2010; Hettich et al., 2014). The estimate then becomes

(6) $${\overset{⌣}{T}}_{g}=m_{up}^{n}g{\tilde{h}}^{n}\overset{⌣}{\alpha_{bs}^{n}}$$

where the expression $\overset{⌣}{\alpha_{bs}^{n}}$ = *atan*2($\overset{⌣}{{COM}_{y}^{n}}$, $\overset{⌣}{{COM}_{x}^{n}}$) represents the angular sway in space of the center of the body mass above the nth joint, while ${\tilde{h}}^{n}$ is the average height of the COM of all the segments above the controlled joint.

#### Support surface linear acceleration

In the presence of support surface acceleration described in the reference system of the support, an inertial force on the center of mass of the body arises. The external acceleration is computed for each joint. The part of the vestibular head acceleration signal not explained by trunk rotation at the hip or at any joint below is taken to stem from support surface acceleration. This is expressed as

(7) $$\alpha_{EXTERNAL}\;=\;\alpha_{VESTIBULAR}-\alpha_{SELF}$$

where the acceleration produced by the joint movements is:

(8) $${\overset{⌣}{a}}_{SELF}^{n}={\overset{⌣}{a}}_{SELF}^{n+1}+L_{n}\frac{d^{2}}{dt^{2}}\left[\begin{array}{c} sin({\overset{⌣}{\alpha }}_{ls}^{n})\\ cos({\overset{⌣}{\alpha }}_{ls}^{n}) \end{array}\right]$$

The disturbance *inertial torque* then results from

(9) $${\overset{⌣}{T}}_{in}=\;{\overset{⌣}{a}}_{EXTERNAL}{\overset{⌣}{\mathrm{COM}}}_{\;}^{n}m_{up}^{n}$$

For simplicity, here only the horizontal translation is considered

(10) $$T_{in}={\ddot{x}}_{EXTERNAL}COM_{y}^{n}m_{up}^{n}$$

#### Contact force disturbance (such as a push or pull)

Humans may sense amount and location of a force exerting impact on the body directly (locally), and in addition may sense the impact that the force has on their balancing in terms of the evoked center of pressure (COP) shifts under the feet, which is proportional to ankle torque. Recall the SIP case scenario of Figure 1, where the external torque *T*_*ext*_ affects the sensory measure of the actively produced torque (*T*_*a*_). It contributes together with the gravitational torque, inertial torque, passive torque (from muscle and connective tissues) and total torque (*T*_*g*_, *T*_*in*_, *T*_*p*_, and *T*_*A*_, respectively) to *T*_*ext*_, which thus is given by

(11) $$T_{\text{ext}}=T_{A}-T_{g}-T_{in}-T_{p}-T_{a}$$

The term *T*_*A*_ is computed using the down-channeled signal $\overset{⌣}{\alpha_{bs}^{n}}$ and, $J_{up}^{n}$, the moment of inertia of the supported body segments as

(12) $$T_{A}=\frac{d}{dt}\left(\frac{d\overset{⌣}{\alpha_{bs}^{n}}}{dt}\;J_{up}^{n}\right)$$

This expression takes in account that also $J_{up}^{n}$ may change with respect to time due to the movements of other DoF. Similarly to $m_{up}^{n}$, also $J_{up}^{n}$ is computed down-channeling information through the modules. For simplicity the down-channeled value is the moment of inertia computed as

(13) $$\begin{array}{l} \tilde{J_{up}^{n}}=\tilde{J_{up}^{n+1}}+m_{up}^{n}{\left\|{\overset{⌣}{\mathrm{COM}}}^{n+1}-{\overset{⌣}{\mathrm{COM}}}^{n}\right\|}^{2}\;+J^{n}\\ \;+\;m^{n}{\left\|\mathrm{CO}M_{link}^{n}-{\overset{⌣}{\mathrm{COM}}}^{n}\right\|}^{2} \end{array}$$

Where *J*^*n*^ and ${\text{COM}}_{\text{link}}^{\text{n}}$ are, respectively, the moment of inertia and the position of the COM of the nth link. The moment of inertia used in Equation (12) is

(14) $$J_{up}^{n}=\tilde{J_{up}^{n}+}m_{up}^{n}{\left\|{\overset{⌣}{\mathrm{COM}}}^{n}\right\|}^{2}$$

Compensating the estimated *T*_*ext*_ may imply that, in a system with neural time delays, positive feedback in *T*_*a*_ requires a limitation of the compensation (gain < 1, low-pass filtering), similarly to the case of the translation estimator. Model simulations suggest that humans may deal with these flaws by transiently increasing passive stiffness, which has zero time delay, through co-contraction of antagonistic muscles pairs.

##### Servo loop and compensation of external disturbances

The disturbance compensation is implemented by summing the disturbance estimates with the input of the controller (PD) with negative sign for compensation (compare Figure 1). In the case of the support surface tilt this can be seen as a coordinate transformation of the controlled variable from joint coordinates to space coordinates. The other disturbances are normalized by $m_{up}^{n}g{\tilde{h}}^{n}$, which represents an angle equivalent to the torque, i.e., the angle that would produce the torque evoked by gravity during body (or segment) lean in linear approximation. This makes the input of the PD controller homogeneous. The torque commanded by the servo controller in the *n*th module is defined by

(15) $$\begin{array}{l} T_{a}=K_{p}\left[\varepsilon -\left(T_{g}+T_{in}+T_{ext}\right)/m_{up}^{n}g{\tilde{h}}^{n}\right]\\ \;+K_{d}\left[\dot{\varepsilon }-\left({\dot{T}}_{g}+{\dot{T}}_{in}+{\dot{T}}_{ext}\right)/m_{up}^{n}g{\tilde{h}}^{n}\right] \end{array}$$

where *K*_*p*_ and *K*_*d*_ are the proportional and the derivative gain, respectively, and ε is the error of the controlled variable as computed using the up-channeled information

(16) $$\;\varepsilon =\{\begin{array}{cc} \alpha_{ls}^{n}!-\hat{\alpha_{ls}^{n}} & if\;the\;controlled\;variable\;is\;\alpha_{ls}^{n}\;\\ \;\alpha_{bs}^{n}!-\tilde{\alpha_{bs}^{n}} & if\;the\;controlled\;variable\;is\;\alpha_{bs}^{n}\\ \alpha_{lf}^{n}!-\alpha_{lf}^{n} & if\;the\;controlled\;variable\;is\;\alpha_{lf}^{n} \end{array}\;$$

where $\tilde{\alpha_{bs}^{n}}$ is an estimate of the COM sway of all the links above the controlled joint, as computed using the up-channeled variable $\hat{\alpha_{ls}^{n}}$.

The effect of each disturbance input and of the error signal of the servo loop can be adjusted by gains (specified as control module parameters). In contrast, the relation between proportional (*K*_*p*_) and derivative (*K*_*d*_) gain is fixed for all disturbances. Gravity compensation with lasting body lean represents a special case. Modeling of human responses to *slow* support surface tilts yielded better results when using a PID controller instead of a PD controller (Mergner et al., 2003; Peterka, 2003) For force feedback as an alternative for the I, see Peterka (2009). The solution used here is a gain elevation in the gravity estimator for low frequencies (Schweigart and Mergner, 2008). As an alternative, Ott et al. (2016) used a PID controller for the servo in robot experiments and in addition a specific PD controller for each disturbance estimator.

The DEC implementation used in the robot experiments of Ott et al. (2016) included feedback from passive joint stiffness and damping. In humans, passive stiffness and damping amount to ~10% of the active stiffness and damping, stemming mainly from connective tissue properties of muscles and tendons. Having impact with virtual zero latency, they improve control stability in face of the considerable time delays of the reflexive loops (lumped delay ≥100 ms for the ankle joint). Their implementation in the robot experiments of Ott et al. (2016) helped in stabilizing the control, as was the case in our previous experiments with Posturob II (Hettich et al., 2014). Implementation of passive stiffness and damping in the Lucy robot is still pending. Further, the implementation of predicted-sensory estimates with pro-active movements, whose effectiveness has been shown before in Posturob I (Mergner, 2010), awaits implementation in Lucy.

### DEC control in the frontal plane and combined in the sagittal and frontal planes

Conceptually, one DEC control module can be used to control both legs during stance control in the sagittal plane, as realized in our robot experiments in Posturob I and II (Mergner et al., 2009; Hettich et al., 2014). This applies when the two legs and feet are aligned in parallel such that the rotations in the two ankle joints occur approximately around a common axis (for special conditions in which humans weight the proprioceptive input into the controls of the two legs independently of each other, see Pasma et al., 2012). Analogous simplifications hold for the hip joints when extending the control to upper body rotations, and to the knee joints when considering vertical body movements. A mechanically special situation (“four bar linkage”) with only one DoF is given with biped balancing in the frontal plane. In this study, stance width with approximately parallel legs is exclusively considered (Figure 3A). In particular, the torque produced in the frontal plane around the ankle joints is relatively small, but the legs can overall produce a large torque on the support surface and hence on the body COM for body stabilization and movements.

**Figure 4:**
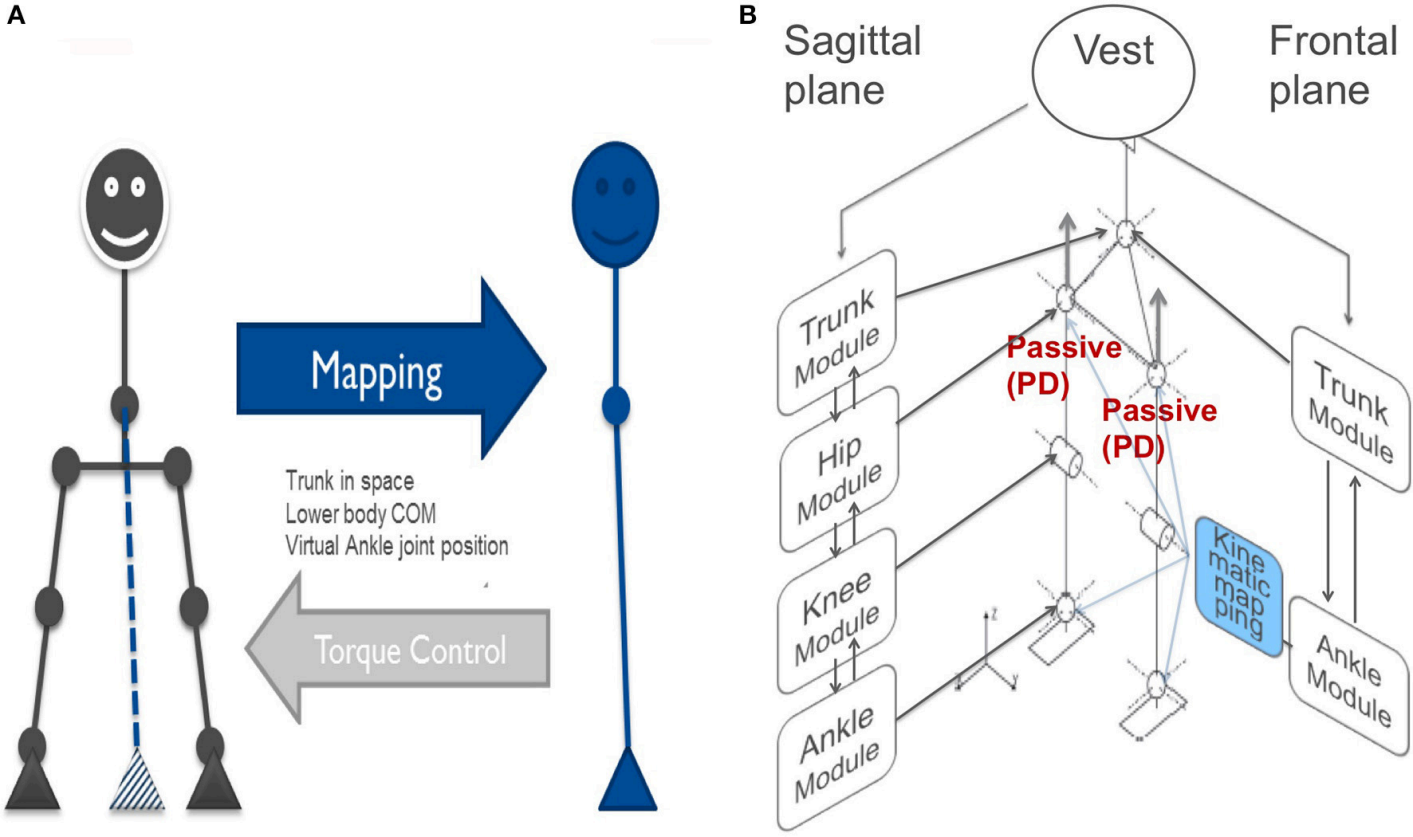
**(A)** Balance control in the frontal plane modeled as a double inverted pendulum. **(B)** For balancing during the squatting experiments (see section Experimental Procedures and Testbed (5), Voluntary Squatting Movements and Figure 10), the control is extended to employ 6 DEC control modules that control 10 mechanical DOFs in the frontal and sagittal planes. Note that horizontal hip control is passive proportional-derivative (PD). This full body posture control can, in principle, be used to start gait. Low loop gain provides mechanical compliance (and keeps energy consumption low).

Inspired by a model interpretation of human balancing in the frontal plane (Goodworth and Peterka, 2010), Lippi et al. (2016) suggested for the Lucy robot a preliminary generalization of the DEC control to the frontal plane, formalizing the body kinematics as a double inverted pendulum. The four bar linkage system with one degree of freedom for the lower body control applies if the knee movements are negligible (true with moderate disturbances) and when the feet are continuously kept in contact with the support surface. Then, a simplified model in terms of the SIP scenario applies to lower body sways about a virtual ankle joint between the two actual feet and can be connected by a virtual link to the pelvis joint (Figure 4A). Body balancing is then achieved in terms of controlling the position of the COM of the whole body in the frontal plane by applying the appropriate torque to the support surface and maintaining the vertical orientation of the HAT segment above the pelvis. The desired torque for the virtual joint is distributed in the robot on the four actuated joints (ankles and hips). Using the control equations of the previous section, two DEC modules suffice for the control in the frontal plane, for simplification, one for the lower body and one for the upper body.

The frontal and sagittal planes are mechanically coupled in the physical robot, as are the intermediate planes between them. Theoretically, the interaction between the planes could be considered problematic in terms of coupling forces if the two planes are controlled independently of each other. *Our hypothesis in this study for the control system of Lucy is that, in face of the low loop gain for the disturbance compensations used in the control, the coupling between the dynamics in the two planes is not critical and should allow us to control the two planes independently of each other*. Independent control in the frontal and sagittal planes based on kinematic synergies has been successfully applied for posture control in a small position-controlled humanoid in earlier studies (Hauser et al., 2007).

In the experiments reported below, Lucy was freely standing while actively controlling its balance in the sagittal and frontal planes using the two sets of DEC control modules: one set for the sagittal plane and the other set for frontal plane, while the controls of the horizontal-plane in the hip joints were “passive” through local joint angle proportional and derivative (PD) “proprioceptive” feedback (indicated in red in Figure 4B).

### Experimental procedures and testbed

Performance of the DEC system was investigated experimentally when it controlled the sensorimotor behavior of the humanoid robot during balancing of upright stance while compensating external disturbances and self-produced disturbances arising from voluntary movements. Voluntary movements were produced by defining a reference trajectory for the specific control variables (see below). During the experiments (Figure 2B), the robot was standing on a 6 DoF motion platform (Stewart platform) in a human posture control laboratory (Mergner et al., 2003). Motions of the robot were recorded by capturing motion signals from the robot's internal sensors (same as used for its control) for comparison between desired and actual poses or movements. The responses to external disturbances were recorded using an external opto-electronic device (Optotrak 3020; Northern Digital Inc.; Waterloo, Canada).

List of experiments performed:

1. *Test*: Balancing of COM during upright stance on periodically tilting support surface (SS)—this tested steady state balancing performance.
  *Disturbance:* Sinusoidal SS tilts of peak-to-peak (pp) 2° at 0.1 and 0.2 Hz were applied in three trials: (a) sagittal plane, (b) frontal plane, and (c) 45° with respect to the sagittal and frontal planes.
  *Aim:* Demonstration of stable balancing in all three planes, with cooperative effects for *c* from the simultaneous balancing in the sagittal and frontal planes.
2. *Test:* Balancing of COM during upright stance as in (1), but with transient tilts of the SS. This experiment allowed us to distinguish between static and dynamic balancing performance.
  *Disturbance:* SS tilts with raised cosine velocity profile (dominant frequency, 0.2 Hz) of peak-to-peak 4° were applied again in three trials: (a) in the sagittal plan, (b) in the frontal plane, and (c) 45° with respect to frontal and sagittal planes.
  *Aim:* Evaluation of static and dynamic balancing performances in the sagittal and frontal planes and their cooperative effects occurring in the intermediate plane.
3. *Test:* Balancing of COM during upright stance while pseudo-randomly tilting the SS using the PRTS stimulus^1^. This stimulus allowed us to describe the balancing behavior in terms of FRFs.
  *Disturbance:* Support surface tilts of peak-to-peak (pp) 1°, 2°, 4°, and 8°.
  *Disturbance waveform:* PRTS^1^ (0.016–2.2 Hz), applied again in the sagittal, the frontal, and the 45° intermediate planes.
  *Analysis:* Spectral analysis of the angular excursions of the body COM in space.
  *Aim:* Demonstration of stable balancing in the sagittal and frontal planes across a broad spectrum of tilt frequencies and the cooperative effects occurring between the balancing in the two planes. Varying stimulus amplitude would allow us to test for the human-like non-linearity of responses (expected from the velocity threshold in the ankle module, see Equations 1 and 2).
4. *Test:* Voluntary rapid full body movements in the intermediate sagittal-frontal plane.
  *Movement command:* Starting from a leaning body COM position in an intermediate plane with 45° orientation with respect to sagittal and frontal planes, a fast voluntary movement was commanded to reorient the COM into the vertical position above the feet, using step function references for all the commanded DoFs. The movement was repeated six times to observe the variability of the response. A similar movement was also performed in the frontal plane and in the sagittal plane separately. This allowed us to observe how the coupling effects are affecting the dynamic response in the two planes.
  *Aim:* Demonstrating proactive movements and testing the robustness of the system in face of strong self-produced disturbances including coupling effects between different joints and between the two controlled planes.
5. *Test:* Squatting movements (knee bending).
  *Movement and task commands:* Raised sinusoids with 4° amplitude at 0.17 Hz were used for commanding knee-bending in repetitive cycles from and back to straight. With the instructed tasks of COM balancing in the ankle and hip joints, the commanded knee-bending was associated with reactive compensatory movements of the whole body in the ankle joints and of the upper body in the hip joints. The task of the pelvis-HAT joint was to maintain a vertical HAT orientation in space.
  *Aim:* This test challenged the robot's postural stabilization in that all DoFs in the sagittal and frontal planes were interacting. Demonstrating postural stability with the “emerging” movements of the whole body in the ankle joints and of the upper body in the hip joints were secondary aims of the test. For corresponding experiments restricted to the sagittal plane in a 3 DoF DEC implementation in the TORO robot of DLR, see: https://www.youtube.com/watch?v=3ALCTMW3Ei4).
6. *Test:* Voluntary full body movement at increasing speed.
  *Movement and task commands:* Voluntary body sway movements were commanded simultaneously in the sagittal and frontal planes (the result was a combined movement in some intermediate plane). The common reference signal followed a sinusoidal function with linearly increasing frequency (i.e., *chirp* signal). The amplitude of the reference signal was set to 1° in the sagittal plane and to 2° in the frontal plane. The frequency range was ~0.2–0.7 Hz.
  *Aim:* This experiment tests the frequency limits of the active body sway in the sagittal and frontal planes of the ankle joints. While the previous tests were performed *within* the margins of the system's stability (to characterize the normal behavior of the system), this test pushes the robot beyond these margins to performance limits.

## Experimental results

In the robot experiments, we investigated how the DEC control mechanisms in the sagittal and frontal body planes interact through mechanical coupling of the robot's body in producing reactive responses to external disturbances applied in the 45° intermediate plane (sections Responses to Sinusoidal Support Surface Tilts; Responses to Raised Cosine Support Surface Tilts; Responses to PRTS Support Surface Tilts). A further experiment investigated this interaction for rapid voluntary full body lean movements in the intermediate plane, which were generated by commanding combined action in the sagittal and frontal planes (section Commanded Fast Full-Body Movements in the Intermediate Plane). A fifth experiment (section Voluntary Squatting Movements) tested control stability across all DoFs of Lucy during commanded squatting.

### Responses to sinusoidal support surface tilts

**Figure 5:**
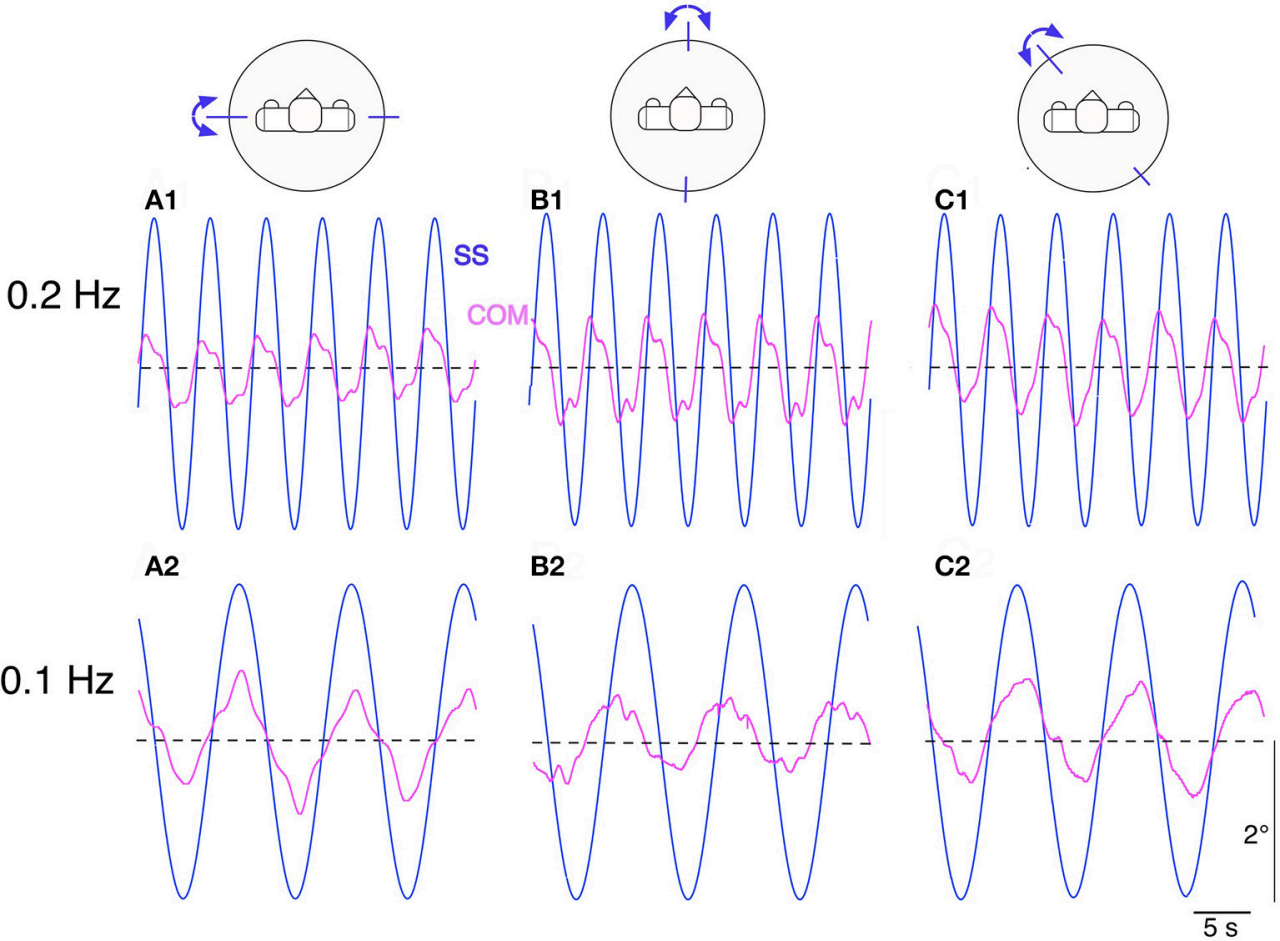
Lucy's sway responses are balancing its body COM during sinusoidal support surface tilt stimuli of ±2° about horizontal. The stimuli were applied at stimulus frequencies of 0.2 Hz and 0.1 Hz in the sagittal plane **(A_1_,A_2_)**, frontal plane **(B_1_,B_2_)** and in an intermediate (45° diagonal) plane **(C_1_,C_2_)**. All sway responses show some under-compensation (body slightly sways in direction of support surface tilt). The responses show slow fluctuations, stemming mainly from vestibular noise (but never showed lasting drifts that might have led to loss of balance). See text for further details.

Lucy's steady state postural responses in terms of body COM sway evoked by the sinusoidal support surface tilts are shown in Figure 5. The responses to the ±2° tilts in the sagittal and frontal plane at 0.2 Hz (Figures 5A_1_,B_1_) and 0.1 Hz (Figure 5A_2_,B_2_) are compared with corresponding responses in the intermediate 45° plane (Figures 5C_1_,C_2_). Note that compensation is similarly stable in all three stimulus planes, with some residual COM lean resulting in the direction of the tilt. This under-compensation is “human-like,” stemming mainly from imperfect support surface tilt estimations in the DEC modules with gain < 1 and velocity threshold (see notches around maxima and minima).

### Responses to raised cosine support surface tilts

**Figure 6:**
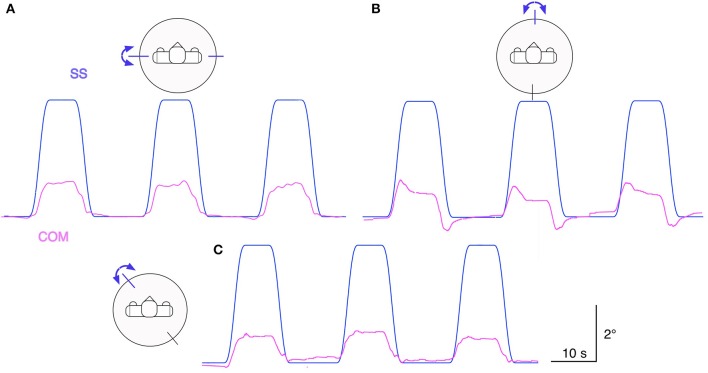
Lucy is balancing its body COM during support surface tilt stimuli with “raised cosine velocity” waveform (4° excursions from horizontal). The tilt was applied in the sagittal plane **(A)**, frontal plane **(B)**, and in the intermediate diagonal plane (45° degrees from the frontal) **(C)**. The robot's responses are slightly under-compensating the disturbances (compare Figure 5). (Waveform resembles that of most human voluntary movements; it is derived from a bell-shaped velocity profile, *v*(t) = –*A* · *f* · cos(2π*ft*) + *A* · *f*, where *t* is time, *A* is angular displacement, and *f* is dominant frequency).

Figure 6 shows Lucy's COM transient and static sway responses in the sagittal, frontal, and intermediate plane to support surface tilt stimuli with raised cosine velocity profile and amplitude of 4° with respect to the horizontal. Dynamic and static response components reflect under-compensation of the tilt stimulus similarly as observed in Figure 6. The responses in the intermediate plane approximately reflect the sum of the responses in the sagittal and frontal planes. The finding of larger dynamic responses in the sagittal as compared to the frontal plane mainly reflects a stronger effect from body inertia. We can further note an absence of static error when the support surface has moved back to the horizontal position, owing to properties of the support surface tilt estimator with velocity threshold and leaky integrator (see Equation 1).

### Responses to PRTS support surface tilts

**Figure 7:**
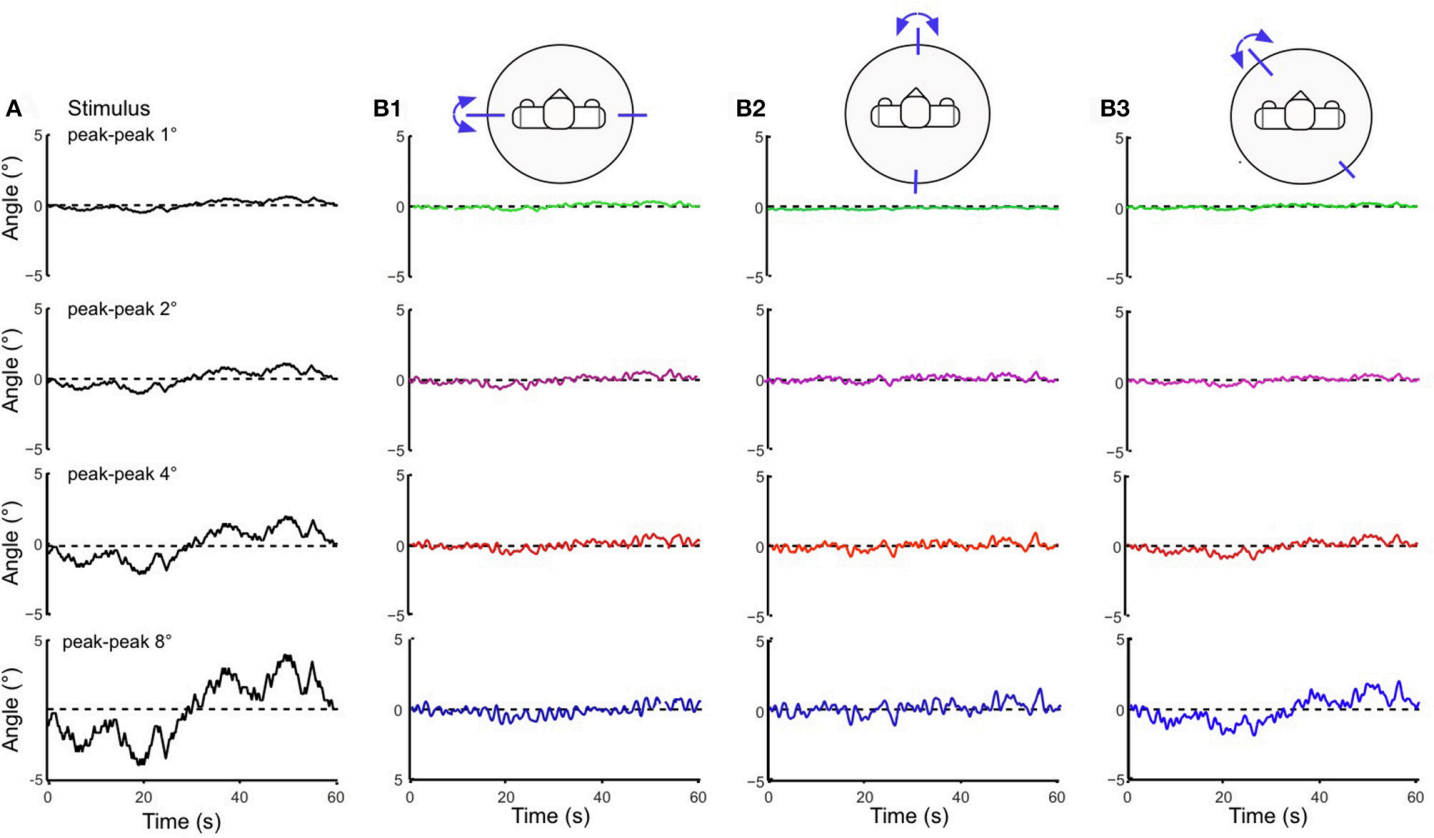
Body COM sway responses of Lucy to PRTS support surface tilts of peak-peak amplitudes of 1–8° **(A)** presented as averaged (*n* = 6) time series (**B1** sagittal plane, **B2** frontal plane, and **B3** intermediate plane). Note that the shown responses represent under-compensation of the stimuli, which is relatively more pronounced for small than for large stimuli (compare gain curves in Figure 8).

**Figure 8:**
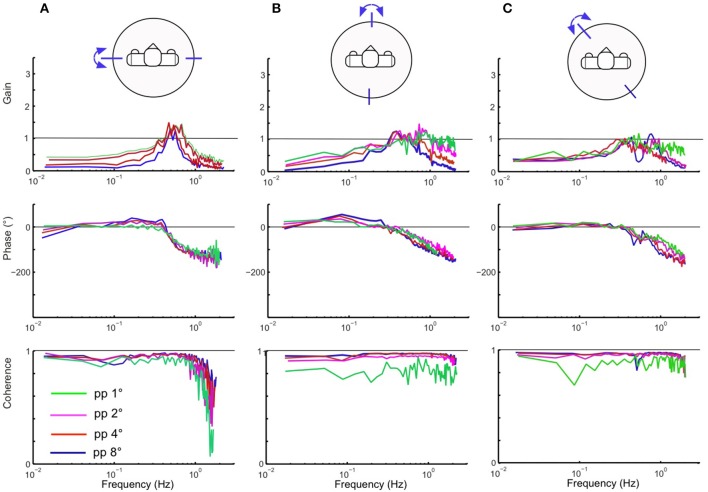
Lucy's sway responses in the sagittal, frontal and intermediate planes **(A–C)** in terms of frequency response functions (FRFs) and coherence functions (bottom) from response averages of 6 repetitions of the PRTS stimulus. Shown are gain, phase and coherence curves over frequency for the four indicated peak–peak stimulus amplitudes. Gain of zero would indicate ideal tilt compensation, a gain of unity that the evoked body COM excursion equals the tilt excursion. The phase gives the temporal response to stimulus relation. Coherence is a measure of the frequency dependent signal-to-noise ratio. Note non-linearity of gain curves (due to the threshold applied to $\overset{⌣}{{\dot{\alpha }}_{fs}^{n}}$) in **(A,B)**, less so in **(C)**.

Testing the robot with the PRTS stimulus allowed us to more comprehensively characterize the frequency and amplitude behavior of the system. Being composed of several velocity step functions, this stimulus has a power spectrum with significant components over the whole range of the frequencies that are of interest here to describe the system dynamics [compare section Experimental Procedures and Testbed (3)]. The stimulus was applied with different amplitudes (pp 1, 2, 4, and 8°) for the support surface tilts in the robot's sagittal, frontal, and intermediate body plane. Lucy's PRTS responses in terms of time series data are shown in Figure 7. They again show under-compensation as in the previous experiments with the sine and raised cosine stimuli. Here, the under-compensation exhibits a non-linearity upon increase in stimulus amplitude. The non-linearity stands out better in Figure 8 where the responses are expressed in the upper panels of the corresponding FRFs as sway (error) gain (zero with full compensation and unity if body motion equals platform motion). Note that gain in Figures 8A,B decreases with increasing stimulus amplitude, being lowest with the pp 8° stimulus—again as can be expected from the velocity threshold contained in the support surface tilt estimation. The basic feature in terms of gain, phase, and coherence resemble each other across the tests in the three body plane tested (Figures 8A–C). Interestingly, we observe a smaller amplitude non-linearity for low frequencies in the intermediated plane (Figure 8C; also compare across in Figures 7B1–B3). Again, we can observe that the coupling between the two planes does no harm to the stability of the system.

### Commanded fast full-body movements in the intermediate plane

**Figure 9:**
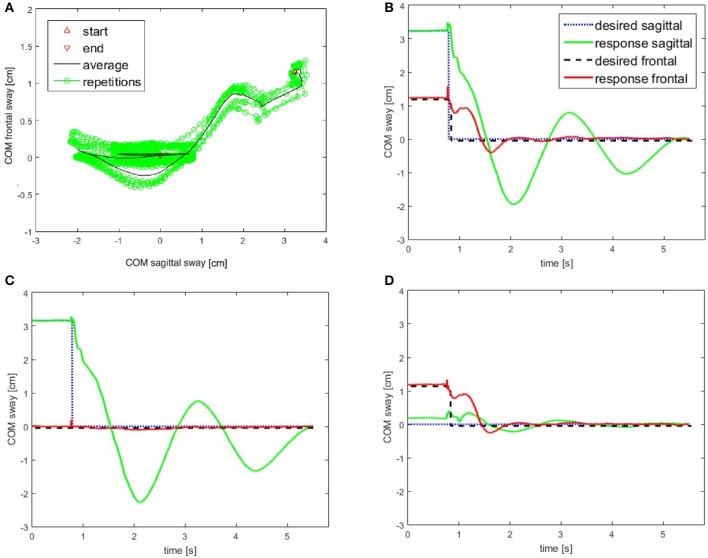
**(A,B)** Return of body COM back to primary position in the course of repeated rapid voluntary movements of the COM in intermediate plane. Starting from an eccentric position (2.3 cm forward, 1.7 cm on the right), the robot was commanded to perform a rapid return of the COM to the upright position. The system response is shown in **(A)** and the COM trajectories are shown in **(B)** (for commands, see profiles of dashed and dotted step functions). **(C,D)** In order to visualize the coupling effects between the two body planes, additional experiments were performed where the robot moved only in the sagittal plane **(C)** and only in the frontal plane **(D)**. Note that all plots are displayed with the same scales to facilitate the comparisons. The control was always active in both planes to keep the system stable.

This test challenged the robustness of the modular control in face of rapid self-produced movements in the sagittal-frontal 45° intermediate plane. The movement is associated with strong disturbances acting mainly through coupling forces arising between most of the robot's controlled DoFs. The test may be critical in a system with distributed modular controls that have time delays. Figure 9A shows the path of the COM sway in the two planes from the starting position at the right upper corner back to primary position (coordinates x = 0 cm, y = 0 cm). The complex path of the return reflects differences in the system's control and mechanical compliance in the two planes. The Figure 9B reports the temporal relaxation in the frontal plane (red) and the sagittal plane (green) following the step. The panel shows decaying oscillations of the relaxation. In the frontal plane, the rise time amounted to 0.584 s, the settling time to 5.115 s, and the overshoot to 33.2% of the step with a peak time of 1.620 s. The corresponding values for the sagittal plane were 0.621 s rise time, 5.141 s for the settling time, and an overshoot of 60.3%. This experiment shows differences in the dynamics in the two body planes, with larger oscillations in the sagittal plane due to higher compliance in this plane. Overall, it demonstrates stable performance when the control and mechanics in the robot's sagittal and frontal planes are dynamically interacting in rapid movements.

In the additional experiments shown in panels Figures 9C,D, the movement was restricted to the sagittal and frontal plane, respectively. They demonstrate that a rapid movement in one plane has only a small effect on the COM in the other plane. The cross talk from the sagittal to the frontal plane is again clearly larger than *vice versa*. Note that the responses in Figures 9C,D are similar to those shown in Figure 9B with respect to the actively moved component. This suggests that the controllers efficiently compensate the disturbance produced by the coupling between the planes. Note also that in all three experiments (Figures 9A–C) the control was active in all the DoFs of the sagittal and frontal planes in order to keep the body upright.

### Voluntary squatting movements

**Figure 10:**
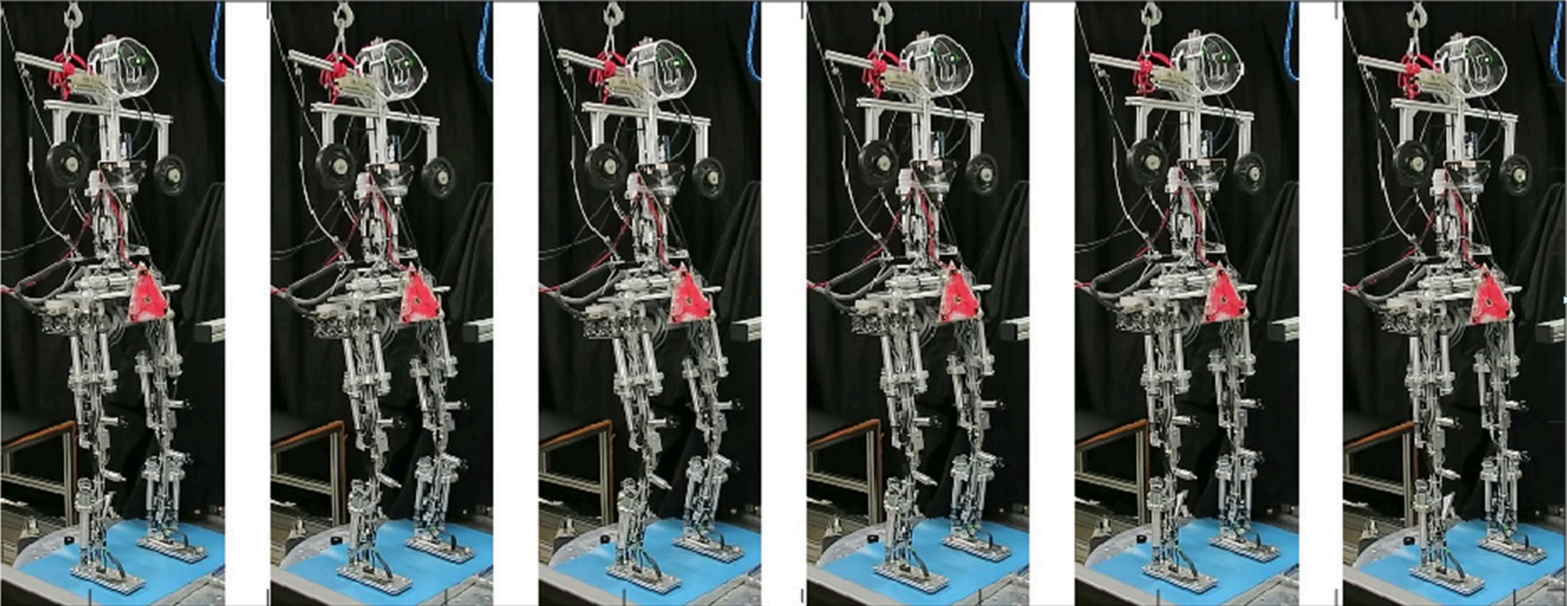
Lucy performing squatting movements. The robot was commanded to perform knee bending at 0.17 Hz while balancing to keep the body upright. The observed coordination between knee, ankle, and hip movements (in terms of compensatory bending in hip and ankle joints) is a property emerging from the interaction between the control modules during the squatting movements (see text).

In this experiment, Lucy performed repetitive squatting knee-bending movements with reactive balancing movements occurring mainly in the ankle and hip joints [see section Experimental Procedures and Testbed (5)]. This experiment challenged Lucy's movement and balancing performance in a situation where all DoFs in the sagittal and frontal planes were interacting. The robot successfully performed this test (see film sections in Figure 10 and film in Supplementary Materials). Lucy executes the voluntary and reactive movements in the sagittal plane, while the frontal plane controls prevent falling by keeping the body upright. No kinematic synergy is imposed explicitly and the resulting joint configurations are produced by the interactions between the modules.

### Voluntary full body movement at increasing frequency

**Figure 11:**
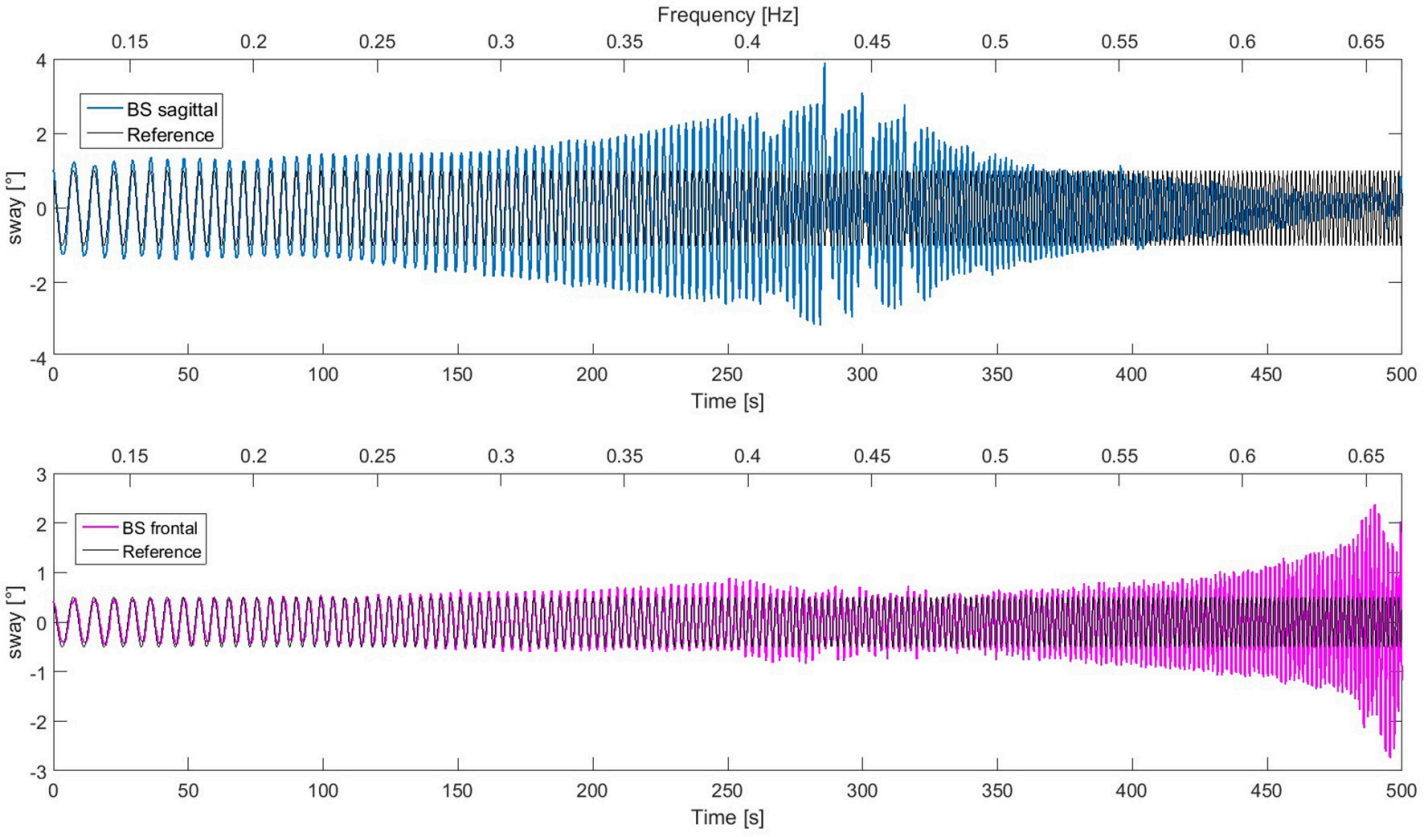
Lucy performing a body sway in the ankle joints in the sagittal and the frontal plane simultaneously at increasing frequency (indicated at top of panels; time at bottom). The responses in the sagittal plane show a peak around 0.42 Hz. For higher frequencies, response amplitude tends to decrease. The response in the frontal plane increases markedly above 0.6 Hz. The recording was finished (right boundaries) when the robot's feet started to lose contact with the support surface due to large oscillations in the frontal plane. The difference in the dynamic responses in the two planes is mainly due to the body mechanics, i.e., the different size of the support base and the different mass distribution (which humans may account for by adjustments in the control, as future work may show).

In this experiment, Lucy performed voluntary body sway movements in the frontal plane and in the sagittal plane simultaneously. The reference trajectory was a sinusoid with increasing frequency (*chirp* signal) with the amplitude of 1° in the frontal plane and 2° in the sagittal plane. In contrast to the previous experiments, which were aimed to characterize the behavior produced by the DEC control within the margins of stability of the system (frequency range similar to that used in human posture control experiments), this trial was performed to the limit of failure (until the robot's feet lost contact with the support surface). The resulting movements are shown in Figure 11. In the low frequency range up to 0.4 Hz, tracking performance was almost accurate in both planes. With further increase in frequency, the responses in the sagittal plane develop a peak and then get smaller, while the responses in the frontal plane remain essentially similar as before and then increase in amplitude, before the response becomes unstable at 0.65 Hz, where the robot lost contact with the support surface. Thus, notably, this task pushed the robot to its stability limits. This owed mainly to limitations of the actuators, which were not designed for high frequencies and torques (which also would exceed human capabilities). Interferences between the sagittal and the frontal plane are relatively small, which supports our notion that the two planes of the robot's body can be controlled by two independent control systems.

## Discussion

The main aim of our neurorobotics approach is to investigate whether the networks of DEC modules so far used separately for the sagittal plane and frontal plane (Lippi et al., 2016; Ott et al., 2016) would adequately cooperate with each other without using a supervising full body model or some other form of software linkage between the two networks. In particular, we asked whether the mechanical coupling between the planes given by the physics of the robot's body would provide postural stability when the robot performs movements in the 45° plane intermediate to the sagittal and frontal planes, or when it balances external perturbations in this plane. We envisaged that the underlying DEC with both voluntary movements and postural reactions follows the rules of vector decomposition from the intermediate into the sagittal and frontal planes. In the experiments 1–5, the robot performed well within the stability margins based on the mechanical properties of the robot and the dynamics of the controls in the two planes, as ascertained empirically also in experiment 6 (Figure 11).

The described experiments and results considerably contribute to our aim of building a robotic system to further develop our human-derived DEC control with the ultimate goal of mirroring in robot experiments the human sensorimotor functions. This approach is complex and cannot be reached in one step, but requires several steps. The here-described steps comprise the cooperation between control modules in the frontal and sagittal planes of our modular control architecture, the testing of both reactive and proactive movement controls in view of control stability including mechanical aspects, and to provide measures such as frequency responses functions for robot-human comparisons. The current steps are important for further DEC developments that aim to implement in future steps more complex sensorimotor functions such as human-like walking.

The main computational challenge we expected in the present experiments was control stability in face of the feedback time delay of 20 ms, which is still much shorter than the known human time delay. Furthermore, the physical anisotropy of the robot's body in the sagittal and frontal planes represented a challenge. This arises among others from differences in body inertia and length of the base of support between the two planes, as reflected in the different responses to external disturbances and the different dynamic performance in voluntary squatting movements shown in the results section (Figures 5–8, 10). Yet, the robot successfully mastered with the same set of control parameters the disturbance and movement scenarios we applied. Thus, our finding demonstrate that the modular DEC control architecture is able to coordinate the movements across the robot's 14 DoF without signal exchange between the DEC module nets for the sagittal and the frontal planes or some higher order control mechanism. This is reminiscent of the so-called embodied approaches (e.g., Brooks, 1991) according to which the body or other loops through the physical world can mediate interaction effects directly, i.e., without the need of explicit connections at control levels. The frequency responses functions obtained in the present experiments give us a comparison basis for future experiments in robots and humans.

The following discussion firstly considers related issues in humanoid robotics and the relevance for robotic neuro-rehabilitation (section Related Issues in Humanoid Robotics and Relevance for Robotic Neuro-Rehabilitation), then insights for the modeling of the human postural control (section Insights for the Modeling of the Human Postural Control), and finally future steps and developments expected for the extended DEC concept (section Future Steps and Developments Expected for the Extended DEC Concept).

### Related issues in humanoid robotics and relevance for robotic neuro-rehabilitation

Taking human bipedal control in standing and walking as a basis for comparison is an important research topic in humanoid robotics (Torricelli et al., 2014). One reason is that human postural and movement skills are still considered to be superior to those of robots, being more robust and efficient, and also covering a wider range of external conditions (Nori et al., 2014). Another reason is that humanoids acting in the human sphere may profit from human-like sensorimotor behavior when interacting with humans and their world.

Among the various tasks of sensorimotor control, maintaining balance is a *primary task* in the DEC concept as well as a basic rule that is followed in many fields of humanoid robotics. S*econdary tasks* can be performed in parallel with this primary task exploiting the kinematic redundancy of the robot, e.g., by projecting the secondary task into the null space of the Jacobian of the balancing task (Sentis and Khatib, 2005). Care is generally taken to constrain secondary tasks such that they are not conflicting with the balancing task. In the present work, multiple tasks are achieved simultaneously, defined by different control variables for different modules. For example, in the squatting movement (section Voluntary Squatting Movements), the ankle module balances the body, while the knee module performs the vertical body motion and the other modules balance the COM of the supported body segments. In general, each module controls a joint (mechanical or virtual), along with all other segments supported by it. This works under the assumption that the controlled variable is affected directly by the controlled joint angle in that every rotation of the ankle joint produces the same rotation of the COM of the whole body around the ankle. For upright stance, the relation between COM sway and ankle joint is

(17) $$\frac{\partial }{\partial \alpha_{lf}^{1}}\alpha_{bs}^{1}=1,$$

so that the servo controller can use the ankle control torque to control $\alpha_{bs}^{1}$. The dynamic effects of joint movements on the supporting links are here neglected. The integration of tasks in which a joint is controlled in order to produce an effect on the supporting links, e.g., using hip movements to control sheer forces under the feet, is currently still an open issue in the DEC concept.

An often-used balance control based on sensory signals is in humanoid robotics the method of the zero moment point (ZMP) criterion (Vukobratović and Borovac, 2004). It allows for balancing against moderate disturbances, which do not require hand contact or a step. This method has been successfully applied in robots with stiff actuation to adjust actual to desired ZMP, e.g., for walking (Hirai et al., 1998; Sentis and Khatib, 2006). However, many robots nowadays use compliant joints, as is the case with Lucy and the human system. The compliance has advantages for robot-world interactions such as collisions and for robot-human interactions. However, the control of compliant joints is more complex due to higher complexity of the dynamics. On the other hand, an important advantage of compliant actuation based on passive stiffness and damping is its immediate response to impact, starting well before time-consuming sensory feedback mechanisms take action (Haddadin et al., 2007). This is comparable to the immediate passive stiffness “feedback loop” in humans from muscles, tendons, and connective tissues, where previous modeling and robot experiments suggested an improved control stability in face of considerable sensory, neural and muscular time delays (Antritter et al., 2014; Ott et al., 2016). While passive stiffness was implemented in Posturob I and II of this laboratory by using pneumatic muscles and in some experiments springs as tendons (e.g., Mergner et al., 2009), it has not yet been implemented in Lucy. This is one of the aims for future experiments with Lucy (see section Future Steps and Developments Expected for the Extended DEC Concept).

In the DEC-controlled robots including Lucy, compliant behavior and low energy consumption are positive side effects from the low control loop gain, which in humans appears to be mainly related to control stability in face of the biological time delays. Effects of the compliance showed up in the presented experiments in the form of residual body sway following external disturbances as well as overshoot and under-damped dynamics with fast voluntary movements. A particularly important beneficial side effect of using low actuation torques was that the robot's feet did not loose contact with the support surface in the experiments 1–5. The effects that passive stiffness has for reducing impact magnitude and improving energy efficiency has been an issue in actuator design (e.g., Ham et al., 2009) and recently has received considerable interest in humanoid robotics. The method has been combined for example with active compliance modulation in the robot COMAN (Li et al., 2012). In this robot, joint stiffness is modulated in response to an external disturbing force by shifting desired body COM such that the resulting center of pressure (COP) shift compensates for the disturbance. This mechanism is employed in combination with other assisting mechanisms that controlled upper body orientation and energy dissipation. Furthermore, flexible robots such a humanoids with compliant “ankle” and “hip” joint equivalents have been controlled with the so-called Reaction Null Space (RNS) formalisms that aim for a reactionless motion control via a feed forward mechanism and an error compensation via feedback (Nenchev, 2013).

Currently, our experiments do not include large perturbations; this topic remains to be addressed in future work (section Future Steps and Developments Expected for the Extended DEC Concept). For example, explicit inspirations from human balancing research for humanoids often address the “ankle strategy” and “hip strategy” (Nishio et al., 2006). In the “ankle strategy,” ankle torque suffices to produce shifts of the body COP to compensate moderate perturbations, whereas the “hip strategy” becomes involved or dominates when the COP is expected to exceed the limits of the support base for balancing, be these limits determined by the feet or a restricted or compliant support surface. Another limiting factor is the maximal ankle torque. The hip movement then generates horizontal ground forces in order to keep the COM over the base of support (Nashner and McCollum, 1985). These bio-inspired dynamic balance mechanisms have been technically realized in a compliant humanoid robot by Hyon et al. (2007) and other groups. Measures related to COM and COP shifts were found to describe postural stability decision limits with increasing perturbation magnitude in three steps: from (1) “COP balancing” in the ankle joints via (2) “centroidal moment point, CMP, balancing” in the hip joints involving a moment about the COM, up to (3) a rescue step, often resulting in a double support (Stephens, 2007). Simulations showed that human “hip” and “ankle” balancing strategies tend to emerge in the presence of perturbations of different magnitudes from the same optimization criterion (Atkeson and Stephens, 2007). These robotic studies in turn provide inspirations from robotics back to human posture control research.

As stated in section Introduction, an important aspect of the DEC control is its modularity. Modular concepts have been widely applied before to both the analysis of human movement and the control of robots. In general, the concept of modular control implies the subdivision of the control problem into several sub-problems that can be managed by separate control modules. Such modules may interact in different ways, e.g., directly by exchange of signals or through the effects they produce on the system. Control modularity has been defined at different levels of abstraction. For example, in the “behavior-based” control systems several sub-behaviors may operate in concert with each other, organized according to a hierarchical principle (Brooks, 1991). In the context of posture control for biped humanoids, such sub-behaviors can be used to control low-level tasks like joint torque profiles, or to process higher-level issues such as adapting control parameters to contextual situations, or performing specific reactive adjustments. For example, an implementation on a simulated humanoid is shown in Luksch (2010).

Higher-level functionalities can emerge from the complexity of behavior based systems. Recently, the integration of internal representations has been considered in behavior based system research; this allows for a direct solution of issues such as movement planning. It finds its basis in that several tasks in biological systems are considered to rely on internal representations for inverse modeling, forward models and sensor fusion (Schilling, 2011). Internal representations are different from a behavior in that they are not designed specifically as tasks, but instead have a general validity, as e.g., realized in the principle of *mean of multiple computations* (MMC). This method can represent body kinematics independently from the specific task and be generalized to arbitrary joint configurations (e.g., of a planar arm in Schilling, 2011 or of a hexapod in Schilling and Cruse, 2008). The DEC control, on the other hand, is specifically addressing posture control and disturbance rejection. Yet, although its bottom-up control design by the integration of locally acting modules shows interesting emerging properties such as segment coordination, it is not expected in general to cover higher level aspects of motor control. An integration of DEC with the above methods can be imagined, e.g., in a set up where high level processing receives sensory input preprocessed through the DEC sensor fusions and where the high level controller outputs into the DEC posture control, e.g., estimates of disturbances that are self-produced by voluntary movements.

Another modularity concept, the Modular Modality Frame model (Ehrenfeld and Butz, 2013), envisages multiple cross-coordinations between cortical sensorimotor representations for processing states of the body and the body parts and the integration of corresponding multisensory information with Bayesian optimality. Again, this concept addresses probabilistic mechanisms of the body schema for movement planning and commanding, whereas the DEC concept deals with the postural control that, on a lower level, helps movement execution by disturbance compensation. Yet, parallels to the DEC concept possibly exist concerning multisensory processing. Specifically, the DEC concept combines in a first step signals from a variety of transducers to obtain estimates of relevant physical variables. In a second step, from these estimated physical variables estimates then the four disturbances are derived. This two-step transformation of transducer signals into estimates of disturbances occurs without probability weighting. The processing in the DEC involves at least partially known transfer characteristics and noise properties and aims to fulfill the commanded task, using a direct reconstruction of the physical relationship between disturbing forces, joint positions and transducer stimulation.

The uncertainty of the results caused by the sensor noise (mainly vestibular noise; van der Kooij and Peterka, 2011) can partially be mitigated by thresholds (Mergner et al., 2009) and disturbance prediction (Mergner, 2010). Estimated disturbances smaller than a given threshold are excluded from the compensation and the control process. This prevents noise from sensory signals that are not participating in the control from influencing the system. To which extent this approach fulfills optimality criteria remains to be investigated.

Exchange of knowledge and inspirations between the robotic and the human domains is especially relevant when it comes to the use of robotic sensorimotor devices such as exoskeletons for neuro-rehabilitation and assistive devices in the development of new invasive or non-invasive stimulation methods to intervene with human sensorimotor functions and more. Knowing the neural control mechanisms behind human sensorimotor behavior will likely prove to be a key for ensuring an intuitive and safe use of such devices by patients.

A related consideration concerns the observation in the present experiments that the robot was able to balance across a broad range of scenarios and conditions using the same sets of control parameters, while automatically adjusting its motor performance to changes in stimulus modality and amplitude, this in combination with the afore discussed low loop gain, and soft mechanical compliance and low energy consumption. Versatility of the control is instrumental for the sensorimotor behavior in a rich and changing environment, which represents a major and still open challenge in the field of humanoid robotics and robotics-inspired assistive devices. The concept of a modular DEC architecture may provide a realistic solution in face of these challenges.

### Insights for the modeling of the human postural control

The coordination of disturbance compensation observed in our experiments when using support tilts in the intermediate plane resulted from mechanical forces that were produced by the controls for the sagittal and frontal planes. A human skeletal body equipped with sensors, actuators and DEC control modules can be expected to function similarly to Lucy. We take this as evidence that the DEC control can be considered as a valid control concept for human reactive postural control. This even applies if the realization in humans would differ in some respect from that of our robot. Differences may owe to the much larger number of sensory transducer and actuators in humans as compared to the robot, which may reflect the fact that humans are experiencing and producing a richer spectrum of sensorimotor behaviors than currently faced by Lucy.

One may object that here exists a conflict between the DEC concept and the notion of those neuroscientists, who assume that movement planning and commanding at the level of the cortical homunculus (compare section Introduction) entails a full-body control. Note, however, that the cortical motor commands, with few exceptions, do not reach the spinal motor centers directly. Rather, the commands pass through and interact with subcortical structures, mainly the basal ganglia and cerebellum, where they undergo modifications. Thereby, they may better fit to the DEC concept. In this view, one important role of these subcortical structures is to complement the voluntary movement commands by predicted-sensory disturbance estimates. These centrally generated estimates are thought to represent fast and low-noisy predictions of the sensory-derived disturbance estimates evoked by the movements, which they substitute for compensation. This allows for a distinction between self-produced and external disturbances and thus allows compensating both even when they occur in superposition (Maurer et al., 2006; Mergner, 2010).

Although the robot experiments provide no direct insights into human postural control, they may provide valuable support for one or the other hypothetical model when this is tested in robot experiments for “real world” robustness (see section Introduction). This neurorobotics approach will allow neuroscientists and roboticists to compare different models both with respect to specific and general performance criteria (e.g., versatility, failsafe robustness, etc.). For example, a concept of how humans may deal with inter-link coupling forces using Eigenmovements has recently been tested in a robot of this laboratory, where it successfully passed the “real world” test (Alexandrov et al., 2017). The DEC concept provides an alternative solution for the problem of inter-segmental force coupling in terms of a bundle of counter measures, which include the DEC mechanisms for *T*_*ext*_ and *T*_*acc*_ in Figure 1, modulation of passive stiffness and damping, and the use of waveforms for commanded movement trajectories, which are optimized for low acceleration and jerk (compare waveform in Figure 6).

In the previous robot experiments of Hettich et al. (2014), the DEC concept was restricted to the DIP scenario and used for the ankle and hip control estimated parameters, which were directly derived from the modeling of human reactive movements. The control parameters related to mass and COM heights of the robot's segments previously used were adjusted in the present experiments to Lucy accordingly. This does not apply, however, to the lumped time delay. Instead, a delay of 20 ms was used for all joints, which is clearly less than that used previously or and cannot be considered “human-like.” Identified lumped delays for the ankle joint control in our previous experiments were ≥100 ms. We have not yet tested such lumped delays in Lucy, but plan to test them together with a number of yet pending robot implementations of related other issues such as the aforementioned envisaged solution for the inter-segmental coupling forces, the up-channeling of control parameters, and the passive stiffness and damping. A further aim is to replace the concept of a lumped time delay by the biologically more appropriate concept of “short latency reflexes” (local proprioceptive mechanisms with latencies of 20–40 ms) and “long latency reflexes” (responses with latencies of 60–300 ms, which involve volition and intention; compare Mergner, 2010). With the integration of these mechanisms we expect stabilizing effects also for coupling forces and a better dynamic performance in faces of challenges that may occur during walking. This also may apply when horizontal body segment movements are included into the control, such as rapid head movements during gaze shifts (Falotico et al., 2017).

In future experiments, we also will address in more detail the emergence of conflict-free interactions between control constituents, as we observed it in our previous and present robot experiments. It allowed here the superposition of voluntary movements and reactive compensations of external disturbances, but also may allow superposition of two or more disturbances at a time. Compliance to the superposition law may represent an important criterion when judging a given robot performance as human-like.

### Future steps and developments expected for the extended DEC concept

To develop the robotic model of the human sensorimotor system further, we consider the following steps important:

- Installing the ability for an automatic reconfiguration of the control model for certain tasks such as walking. There, it will account for changes in the robot's configuration with the alternation between the double leg support and the single leg support while the other leg swings. Smooth human-like walking in future DEC implementations may furthermore benefit from DEC extensions in terms of whole body coordination, which may includes the upper body, and it will involve integration of an open loop gait pattern (compare Lapeyre et al., 2013a,b).
- Predictions of disturbance estimates through both down- and up-channeling of signals that are produced during *voluntary and reactive* movements—with an expected improvement of control stability from lower noise and shorter time delay as compared to sensor-derived signals.
- Further improvement of control stability by using passive stiffness modulation during contact force and support translation disturbances and during rapid voluntary and passive movements.
- Addition of visual self-motion information to improve the balance performance of Lucy. Improvements of accuracy and noise of vestibular and proprioceptive sensor information through interaction with the visual signal can be expected on the basis of human experiments (e.g., Assländer et al., 2015).
- Superposition of more than one external perturbation (e.g., support surface tilts and contact forces) during quite stance and voluntary movements.
- Implementation of human-derived lumped time delays.
- Attempts to mimic sensorimotor impairments of neurological patients using DEC-controlled robots (compare Mergner et al., 2009, for balance model without vestibular function).

## Author contributions

VL and TM performed the experiment and collected and analyzed the data, performed the computer simulations. Both authors contributed to the interpretation of the data, drafted and critically revised the manuscript and finally approved the manuscript for submission.

### Conflict of interest statement

The authors declare that the research was conducted in the absence of any commercial or financial relationships that could be construed as a potential conflict of interest.

## Footnotes

^1^PRTS stands for pseudo-random ternary sequence stimulus (see Peterka, 2002). It allows evaluation of gain of the disturbance-evoked body excursion and its phase and coherence (e.g., with support surface tilt) over a defined frequency range. Data processing comprises a spectral analysis of the stimulus vs. the body (e.g., COM) angular excursions in space using a discrete Fourier transform. The pseudorandom sequence of the tilts makes them unpredictable for humans. See Hettich et al. (2014) for details of the stimulus used.
